# MEG Source Localization Using Invariance of Noise Space

**DOI:** 10.1371/journal.pone.0058408

**Published:** 2013-03-07

**Authors:** Junpeng Zhang, Tommi Raij, Matti Hämäläinen, Dezhong Yao

**Affiliations:** 1 Key Laboratory for NeuroInformation of Ministry of Education, University of Electronic Science and Technology of China, Chengdu, China; 2 MGH/MIT/HMS Athinoula A. Martinos Center for Biomedical Imaging, Boston, Massachusetts, United States of America; 3 Department of Biomedical Engineering, Chengdu Medical College, Chengdu, China; University College of London - Institute of Neurology, United Kingdom

## Abstract

We propose INvariance of Noise (INN) space as a novel method for source localization of magnetoencephalography (MEG) data. The method is based on the fact that modulations of source strengths across time change the energy in signal subspace but leave the noise subspace invariant. We compare INN with classical MUSIC, RAP-MUSIC, and beamformer approaches using simulated data while varying signal-to-noise ratios as well as distance and temporal correlation between two sources. We also demonstrate the utility of INN with actual auditory evoked MEG responses in eight subjects. In all cases, INN performed well, especially when the sources were closely spaced, highly correlated, or one source was considerably stronger than the other.

## Introduction

Magneto- and electroencephalography (MEG/EEG) are noninvasive brain imaging techniques providing millisecond time resolution [1]. However, estimating the distribution of the sources underlying the MEG/EEG signals is complicated by the ill-posed electromagnetic inverse problem: there exists an infinite number of source configurations that can generate identical MEG/EEG responses. Therefore, appropriate constraints are needed to render the solution unique.

MEG/EEG source localization methods can be divided into two classes: dipole source models and distributed source models [2], [3], [4]. Specifically, the most traditional inverse modeling approach in MEG/EEG is to employ the parametric dipole model. In this method, the data are assumed to be generated by a small number of current dipoles whose optimal location, orientation, and amplitude parameters are found with a least-squares fit, see, *e.g.,* [1], [5], [6], [7]. In another class of dipole based methods [8], [9], [10], [11], sources are found by using one probe dipole source to scan one by one all the possible source positions within the whole brain volume. Plotting the goodness-of-fit at each position will yield (pseudo)images, from which the source localization information is derived. “These “dipole scanning” methods avoids nonlinear global optimization and have therefore attracted increasing attention.

In the present study, we use another division of methods, based on whether a method is adaptive or non-adaptive with respect to the measured data. We thus arrive at a division between model-based and data-driven approaches. The model-based approaches include the minimum-norm estimate MNE [12], noise-normalized MNE [13], FOCUSS [14], dipole LORETA [15], charge LORETA [16], CMOSS [17], SCEA [18], LIPSS [19], 3SCO [20] and NESOI [21]. These methods can also be viewed as non-adaptive spatial filters, since their filter weights are independent of the measurements [22]. In many of these approaches, the noise covariance computed from the measurements is used to regularize the solution or assess statistical significance but this noise estimate is based on data acquired outside the time ranges where the signals of interest are present.

Data-driven approaches belong to the “scanning dipole” class of methods. They can be further divided into subspace-based and adaptive spatial filters. The subspace-based methods [23] include classical MUSIC (MUltiple SIgnal Classification) [24], R-MUSIC [10], RAP-MUSIC [11] and INN, the method introduced in the present paper. RAP-MUSIC is a further development of R-MUSIC while both are variants of classical MUSIC. The adaptive spatial filters, often called beamformers, come in several variants. The most widely used one is the linearly-constrained minimum variance (LCMV) beamformer, which was first employed in radar and sonar signal processing [25] and later adopted to MEG/EEG analysis [8], [9].

Compared with model-based methods, adaptive techniques critically depend on the second-order statistics of the measurements to characterize the spatio-temporal features of the data. Comparisons of various adaptive and non-adaptive methods have shown that adaptive spatial filters can achieve much higher spatial resolution than non-adaptive versions [22], and adaptive spatial filters and MUSIC have higher specificity than non-adaptive spatial filters [26].

However, MUSIC-based methods and beamformers perform poorly if there is a high temporal correlation between sources. In particular, it has been shown that bilateral transient [27], [28] or steady-state [29], [30] auditory evoked responses originating from the temporal lobes are difficult to localize with conventional beamformers. Several methods have been developed to address this problem. One possibility is to use a unilateral subset of MEG sensors for one hemisphere at a time [31]. However, this strategy may result in localization errors due to incomplete removal of the signals originating from the sources in the other hemisphere [29] and is limited to situations where the approximate source locations are known *a priori.* The situation can be improved by using a strategy in which coherently interfering sources are almost completely suppressed by adding null constraints to the lead field matrix of the suppression source region when deriving the beamformer weights [27]. However, this method still depends on *a priori* information about the approximate locations of the coherent interfering sources, which prohibits its use as a general solution. A few other methods have achieved limited success but are still suboptimal [28], [30], [32], [33].

The present study proposes the INvariance of Noise (INN) space for MEG source estimation. INN is based on the fact that modulations of source strengths across the evoked response latency change the energy in signal subspace but leave the noise subspace invariant. This method was introduced for direction-of-arrival estimation for radar and sonar applications [34]; it has not been applied to source analysis of MEG signals before. Here we reformulate the previously presented one-dimensional INN for radar problem to deal with the three-dimensional MEG source localization problem and demonstrate that INN is suitable for MEG source localization. The performance of INN is compared with MUSIC, RAP-MUSIC, and beamformers using both simulations and real MEG data. In this study, INN was applied to MEG data only, although in theory it should be applicable to EEG data as well.

## Methods and Theory

### 1. Subspace-based Methods: MUSIC and RAP-MUSIC

The MEG data 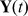 generated by current dipole sources can be modeled as
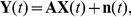
(1)where **A** is the gain matrix relating the measured signals to the dipole amplitudes, i.e., the solution of the forward problem, rows of 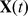 are the time courses of the current dipoles, and 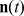 is additive noise.

Assuming that 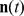 is uncorrelated across the channels, that the variance of the noise on each channel is 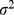, and that the signal and noise are uncorrelated, the correlation matrix of the MEG data is
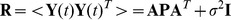
(2)where 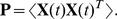


Using the singular value decomposition (SVD) of **R**, we obtain the ordered singular values 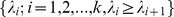 and corresponding singular vectors 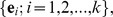 where *k* is the number of MEG sensors. Assuming that the number of dipoles *p* is known *a priori*, we can designate the signal and noise subspaces as 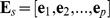 and 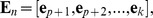 respectively.

Subspace correlation is defined as the set of the the cosines of the principal angles that measure the similarity between the subspaces spanned by the columns of two matrices. The elements in the subspace correlation are ranked in decreasing order, and we denote the largest subspace correlation (*i.e.,* the cosine of the smallest principal angle) of two matrices 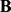 and 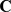 as
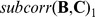
(3)


If 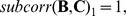 then the two subspaces have at least a one-dimensional subspace in common. Conversely, if 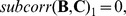 then the two subspaces are orthogonal. The computational methods to obtain 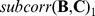 have been described elsewhere [10], [35]. To identify the source locations, classical MUSIC employs the cost function
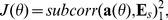
(4)where 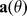 is the signal vector produced by a dipole at location 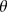.

In practice, the cost function is computed as
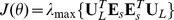
(5)where 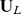 contains the left eigenvectors of 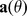, and 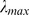 is the maximum eigenvalue of the enclosed expression. Locations of the sources are found as the *p* maxima of this cost function across dipole locations *θ*. One difficulty of this approach is that one must search for multiple local maxima of 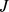 in a 3D brain volume space and such nonlinear searches may miss shallow or adjacent peaks [11].

In RAP-MUSIC, this problem is circumvented by employing a recursive strategy. The first source is found as the global maximum of 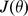. After the first source with parameters 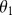 is found, a projection operator
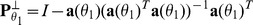
(6)is formed and the next source is found as



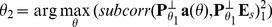
(7)Next, a projection operator including the signal patterns from the two sources already found is applied to find the third source. This process is repeated until all *p* sources are found.

### 2. Adaptive Spatial Filters

Beamformers can be viewed as adaptive spatial filters that pass the signal from desirable locations while blocking signals from other locations. The source activity 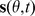 at location 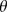 and time 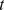 is estimated by a simple linear operation,
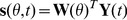
(8)where 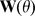 is a column vector consisting of a set of spatial filter weights. In an LCMV beamformer 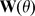 minimizes the variance of the filter output:



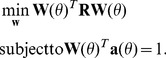
(9)The solution of this constrained optimization problem is [8], [36].
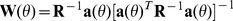
(10)


Mapping the filter output as a function of location generates a functional (pseudo) image. In this study, we use a vector LCMV beamformer described in previous references [27], [36].

### 2. INN

Theoretically, if we increase the source strengths, the variance of the signal subspace will increase whereas distribution of the noise subspace remains the same. In other words, the noise subspace is invariant with respect to the strengths of the sources. INN is designed according to this property.

Let us define a new matrix 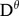 as
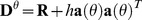
(11)where 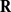 is the data correlation matrix of Eq. (2), 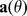 is the field distribution generated by a unit source at location 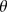, and 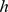 is a positive constant scalar. To balance the order of magnitude between 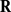 and 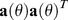, we further rewrite Eq. (11) as,
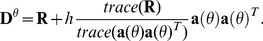
(12)SVD of 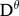 generates the ordered singular values 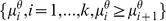 and the corresponding singular vectors 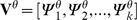. Eq. (12) is the formula that we adopted in the following computation. As 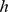 is a positive scalar, it can be proved that [37],
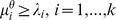
(13)Importantly, when 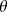 matches one of the source locations, the last 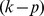 eigenvalues of 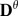 and 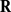 would be the same, i.e.,



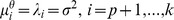
(14)In other words, the energy of the noise subspace obtained from 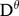 is the same as that obtained from 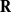.

Note that the property stated in Eq. (14) does not depend on the value of scalar 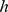 explicitly and will hold for any positive 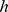. In the simulation section we will study how to select a proper value for 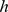. In numerical calculations, because 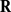 is obtained from a finite number of time instants, the variance of the original and new noise subspaces may not be exactly the same and Eq. (14) does not exactly hold. Thus, in practice, we search the sources in a manner where 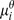 in Eq. (13) not exactly but as closely as possible equals to 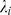. Therefore, an appropriate cost function to consider is:
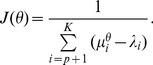
(15)


The values of the cost function 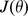 can be used as the imaging index at each grid points within the whole brain volume to generate pseudo-images. The positions where peaks are found are regarded as the locations of the sources.

In summary, the INN algorithm consists of the following steps:

1. Compute 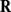
from the MEG data.
2. Compute the SVD of 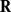.
3. Choose a positive 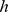 and compute 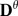
at each possible source location 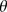 (refer to the following section 3.5).
4. Compute 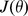.
5. Repeat steps 3 and 4 for all putative source locations.
6. The positions of sources are those where 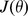 has a local maximum.

Compared to MUSIC, one important advantage of INN is that it is insensitive to strength differences and correlation between sources, even for closely spaced sources. This property is especially important when a weak source is present in the vicinity of a strong one.

**Figure 1:**
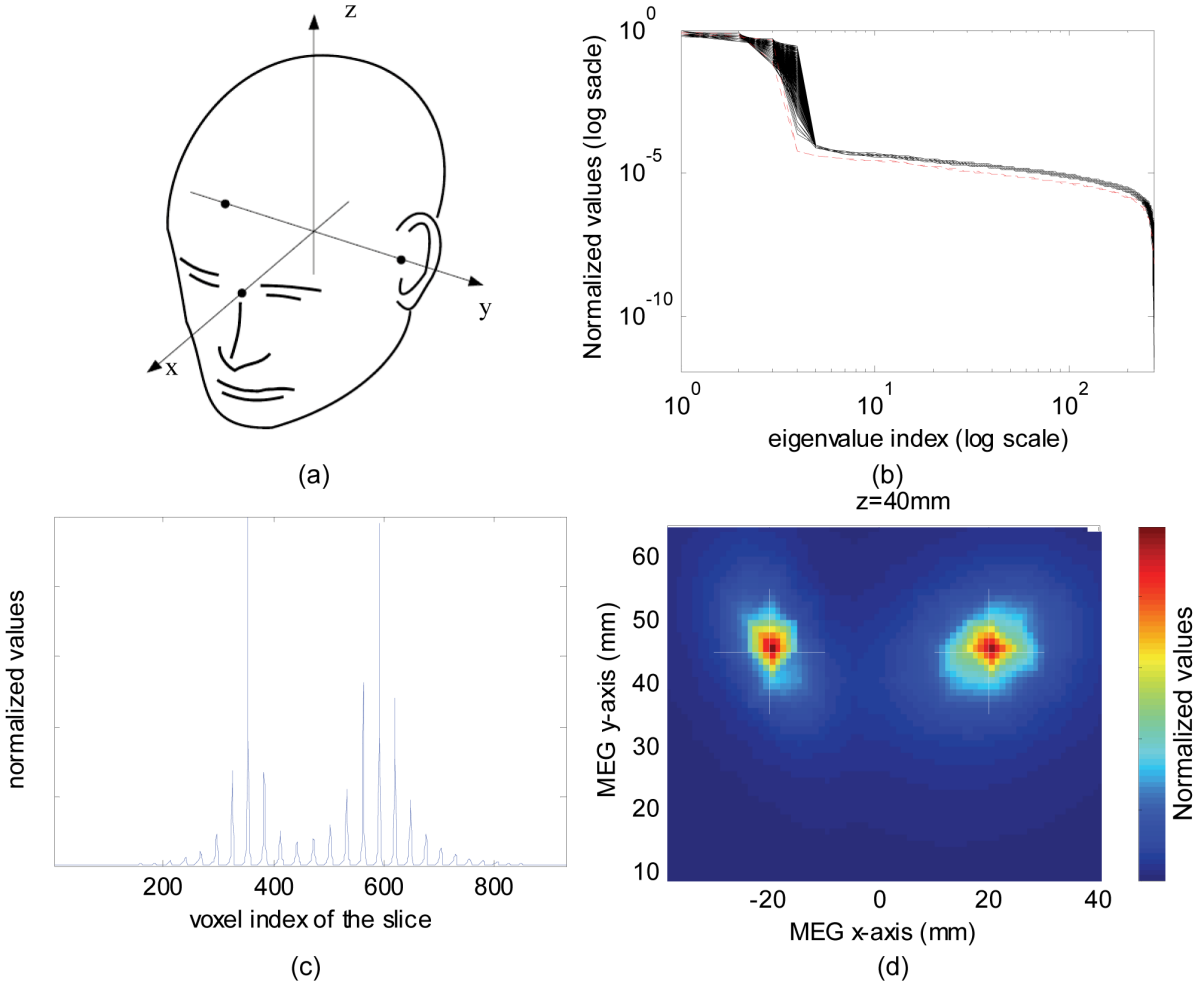
A simulation illustrating the principles of INN. (a) Coordinate system used in the simulations. Two current dipole sources at (−20, 45, 40) mm and (20, 45, 40) mm were simulated in a horizontal slice through the brain (z = 40 mm, 930 voxels, MEG array with 272 sensors). The orientation of the two sources is (−0.6377, 0.3337, −0.6943) and (−0.3876, 1.3302, 0.2834). (b) The curves depict the log scale distribution of the normalized eigenvalue difference of the signal subspace and the noise subspace between the new constructed matrix 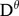 and the original data correlation matrix 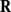 (Eqs. 2 and 11). The two largely overlapping red lines show the distribution for the test source exactly at the two true source locations. The black lines show the distribution at the remaining 928 locations. The fact that the red and black lines are clearly separate when the eigenvalue index is equal to or larger than 4 suggests that INN is able to localize sources. (c) The normalized cost function distribution when the dimension of the signal subspace is 4. At the two simulated source locations (voxel indices 353 and 597) there are two clear peaks, with additional but clearly weaker peaks in neighboring locations. (d) An INN imaging result on the slice at z = 40 mm. The white crosses show the true source locations.

Figure 1 demonstrates the principle of INN in MEG source localization. The definition of the coordinate system used in all simulations is shown in Figure 1a. One horizontal slice through the MEG head model (see simulations section for details) containing 930 grid points, has two simulated sources. Figure 1b shows the log scale distribution of the eigenvalue difference between the new created matrix 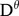 and the original correlation matrix 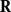 for 930 possible source locations. The two red dashed lines indicate the distribution of eigenvalues when the source location 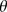 matches the location of the simulated sources, while the overlapping 928 black lines show the distribution of the eigenvalues at the remaining 928 locations. In the noise subspace (eigenvalue indices 4…272), the lines clearly fall into two separate groups, which shows that INN can identify the correct source locations. Figure 1c shows the cost function at all 930 grid points on the slice (z = 40 mm) when the dimension of the signal subspace *p* = 4. There are two clear peaks at the correct source locations with additional but weaker peaks in neighboring locations. Figure 1d shows the imaging results on the slice z = 40 mm.

## Simulations

### 1. Model Configuration and Parameter Definition

In the simulations, the spherically symmetric MEG forward model [38] was employed. The source space had a spherical shape (radius = 90 mm) with a 5-mm spacing between sources. The simulated sensor array comprised 272 magnetometers arranged in a hemispheric array on a sphere with 100 mm radius. The average distance between sensors was 22 mm. The SNR was defined as the ratio of the Frobenius norm of the data matrix to that of the noise matrix. We used correlation coefficient (*r* ^2^) to measure the degree of linear correlation between two source waveforms.

### 2. Resolvability of Closely Spaced Sources

We first tested how correlation and SNR modulate localization accuracy of different methods for closely spaced sources. Two equally strong tangential sources were simulated: dipole 1 was located at (−5, 45, 40) mm with orientation (−0.5797, −0.1380, −0.6621) and dipole 2 was at (5, 45, 40) mm with orientation (−0.9341, 0.1489, −0.3246). The distance of the sources was thus 10 mm. The waveforms of the two sources were 10 Hz sine functions with different phase and 500 ms duration. The sampling frequency was 1000 Hz. The correlation coefficient (*r* ^2^) between the two sources was set to 0.99, 0.7, 0.5, or 0 by adjusting the phase difference between the waveforms. Uncorrelated white Gaussian noise was added to all data points scaled such that SNR was 1.5 or 3. Simulated evoked MEG responses were generated and source analysis conducted separately for each combination of *r* ^2^ and SNR.

**Figure 2:**
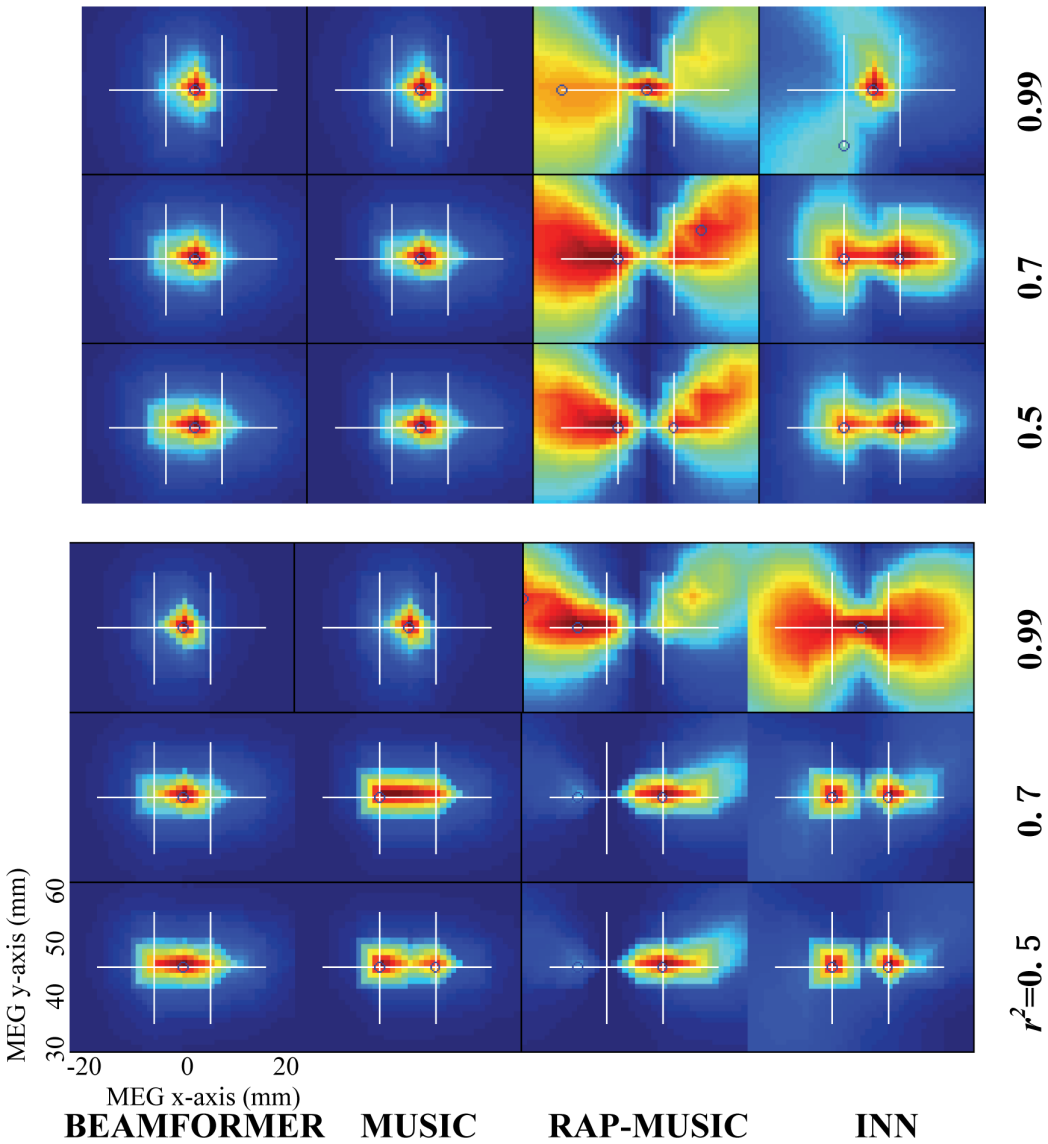
Comparison of BEAMFORMER, MUSIC, RAP-MUSIC (2nd recursion) and INN with two closely spaced (10 mm apart) simulated sources at different levels of correlation (*r* ^2^ = 0.5, 0.7, or 0.99). The true source locations are (−5, 45, 40) mm and (5, 45, 40) mm. SNR is 1.5 (upper panel) or 3 (lower panel). The white crosshairs indicate the true source locations and the blue circles the local maxima found by the different methods. The left bottom corner shows the x- and y-axis scales.

Figure 2 shows localization results of the four methods with varying degree of correlation between the sources and with different SNRs. In the SNR = 1.5 case (upper panel), BEAMFORMER and MUSIC performed poorly for all degrees of correlation. Both of them have difficulty separating the correlated closely spaced sources. RAP-MUSIC also performed poorly; since the first source could not be identified, the second recursion of RAP-MUSIC was incorrect. For moderately correlated (*r* ^2^ = 0.5 and *r* ^2^ = 0.7) sources, INN (*h* = 1) could resolve them accurately. However, for the highest correlation (*r* ^2^ = 0.99), INN, similar to the other methods, had difficulty in resolving the sources, placing a false combined source between the true locations. For uncorrelated sources (not shown), MUSIC and BEAMFORMER resolved the two sources successfully. It should be noted that the SNR = 1.5 is lower than that of typical evoked responses consisting of 100–200 trials resulting in SNR = 5…10. The lower panel of Figure 2 shows the localization results for SNR = 3. As *r* ^2^ decreases, INN resolved the two sources accurately with less spatial blurring. However, BEAMFORMER could not resolve the two sources except for *r* ^2^ = 0 (not shown). As *r* ^2^ was decreased to 0.7, MUSIC started to resolve the two sources, with increasingly clear separation with smaller *r* ^2^ values. RAP-MUSIC (the 2^nd^ recursion) improved in a similar fashion as MUSIC since its performance depended on the MUSIC results in the first iteration.

### 3. Symmetric Sources in the Two Hemispheres

Two spatially separated but correlated sources were placed at (−5, 45, 40) mm and (−5, −45, 40) mm to simulate bilaterally symmetrical sources (same z-plane; 90 mm apart) corresponding to activity in the left and right auditory cortices. SNR was set to 0.5, 0.8, 1, or 2, and *r* ^2^ was adjusted to 0.5, 0.9, or 0.99 by varying the relative phases of the waveforms.

**Figure 3:**
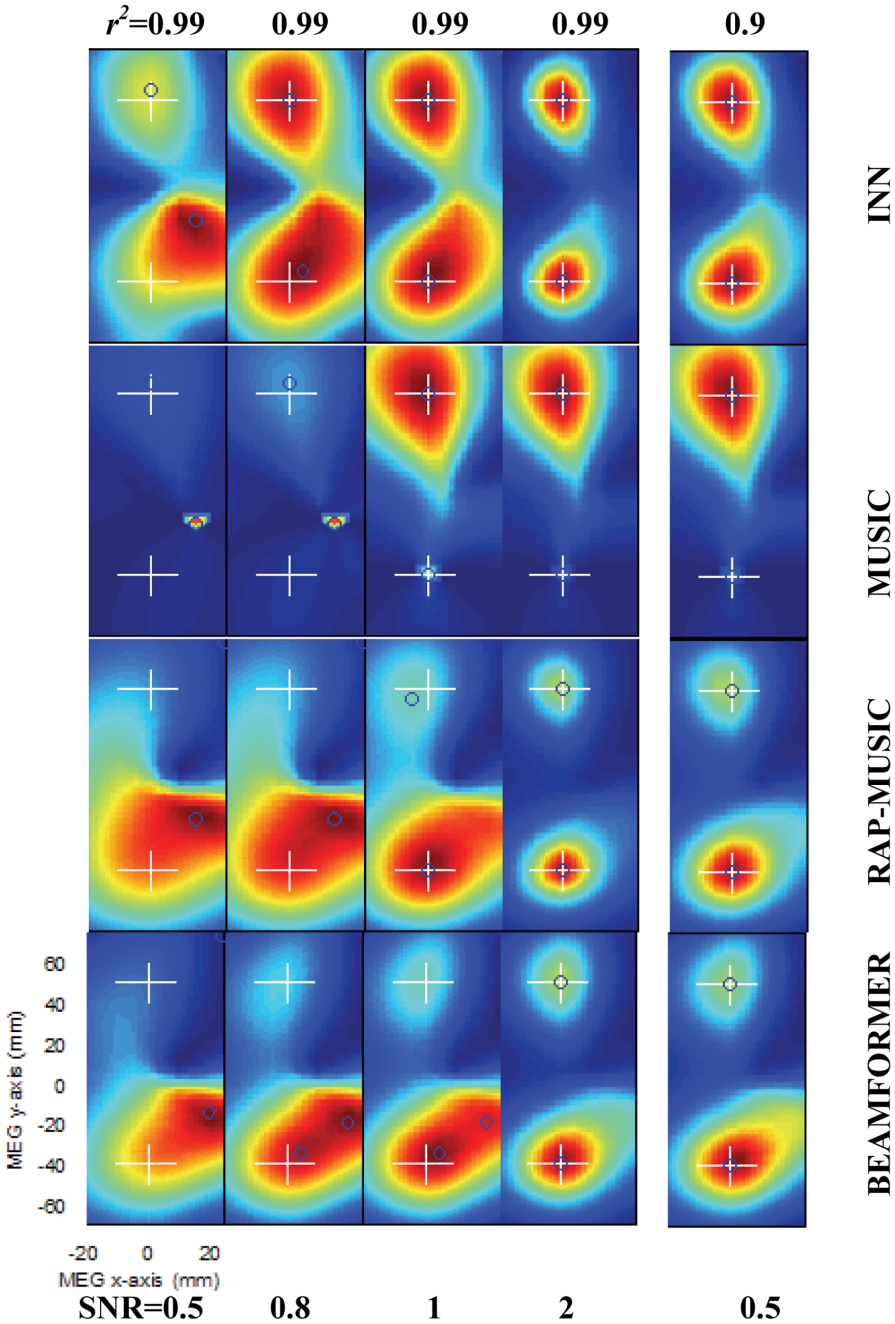
Comparison of BEAMFORMER, MUSIC, RAP-MUSIC (2^nd^ recursion) and INN with two widely (90 mm) separated simulated sources at multiple SNRs and correlation coefficients. The true source locations are (−5, 45, 40) mm and (−5, −45, 40) mm. In the first four columns SNR varies at 0.5–2 and *r* ^2^ = 0.99; in the rightmost column SNR is 0.5 and *r* ^2^ = 0.9. The white crosshairs indicate the true source locations and the blue circles the local maxima. The left bottom corner shows the x- and y-axis scales.

Figure 3 shows localization results for the four methods in a subset of the SNR and *r* ^2^ values. For INN, we selected *h* = 1. When *r* ^2^ was fixed at 0.99, with increasing SNR the performance of all four methods improve gradually (columns of Figure 3). As expected, BEAMFORMER and MUSIC results were similar. For BEAMFORMER, MUSIC and RAP-MUSIC, a spurious additional source disappeared at SNR ≥2. When *r* ^2^ was reduced from 0.99 to 0.9, all four methods identified both sources, although one of the peaks in the cost function was clearly shallower than the other in BEAMFORMER and MUSIC (rightmost column). In other cases (*r* ^2^≤0.9 and SNR ≥0.5), the localization performance (not shown) was similar to that shown in the last column of Figure 3. In all cases, based on visual inspection INN had the best performance.

### 4. Sources with Large Strength Differences

This simulation was designed to study the performance of the four methods for two closely separated sources with a large strength difference. The source locations and waveforms were identical to those used in Section 3.2, except that the strength of one source was five times larger than the other. At SNR = 3, the phase difference between the two sources was adjusted for obtaining correlations of *r* ^2^ = 0.3, 0.8, 0.95, or 0.99.

**Figure 4:**
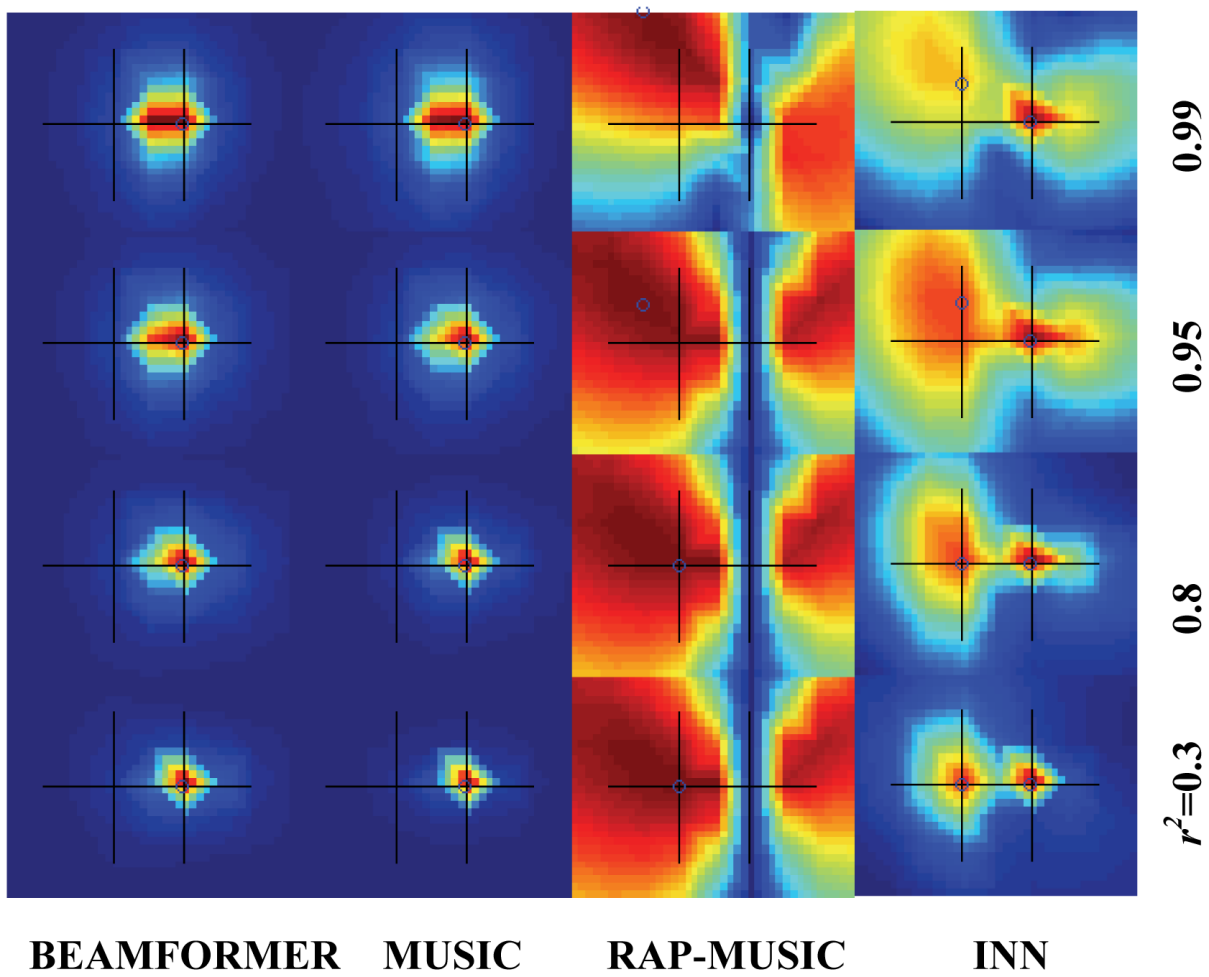
Comparison of BEAMFORMER, MUSIC, RAP-MUSIC (2^nd^ recursion) and INN with two simulated sources with a large strength difference (the posterior source is 5 times weaker than the anterior one). The true source locations and the xy-scales are the same as in Fig. 2 (10 mm distance between the sources). SNR is 3. The two sources were correlated with a correlation coefficient of 0.3, 0.8, 0.95 or 0.99. The black crosshairs indicate the true source locations and the blue circles the local maxima.

Figure 4 shows the corresponding localization results. Again, for INN, *h* = 1. For all cases, BEAMFORMER and MUSIC only identified the stronger source. With a higher correlation, the pseudo-images became more and more spatially blurred. In almost all cases, RAP-MUSIC identified the weaker source with spatial blurring, while the stronger source was suppressed. RAP-MUSIC results show a long clear ditch from top to bottom, which reflect suppression of the stronger source found in the first recursion. With *r* ^2^ = 0.99, RAP-MUSIC failed since the maximum of MUSIC was located halfway between the two sources. INN was able to correctly locate both sources at low *r* ^2^ values, but with a higher correlation, the two sources became more blurred and finally fused together at *r* ^2^ = 0.99. Note that the left (weaker) source appeared more diffuse than the right (stronger), especially when the two sources were highly correlated.

### 5. Selection of the *h* Parameter in INN

This simulation was conducted to investigate the effect of parameter 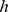 on INN source localization. Simulation parameters were the same as in Section 3.2. At each combination of SNR and *r* ^2^, 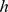 was parametrically varied to 0.001, 0.01, 0.1, 1, 10, 100, 1000, 10^5^, 10^10^, or 10^15^. A simulated evoked MEG response was generated separately for each combination of SNR, *r* ^2^, and *h*. SNR was set to 3. The two sources were correlated with *r* ^2^ = 0.30 or *r* ^2^ = 0.90.

**Figure 5:**
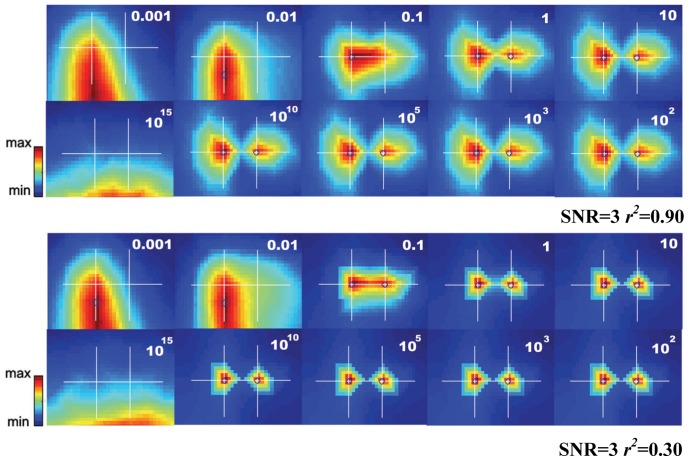
INN images for two simulated closely spaced sources as a function of parameter 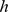. The original source locations and the xy-plane axis scales are the same as in Figure 2 (10 mm apart). SNR was set to 3. The two sources were correlated with a correlation coefficient of 0.90 (upper panel) or 0.30 (lower panel). The 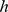 value used in each profile is shown in the upper right corner of each panel.

Figure 5 shows the INN results for *r* ^2^ = 0.30 (upper panel) and *r* ^2^ = 0.90 (lower panel) at increasing *h* values. With both very small (≤0.1) and very large (10^15^) *h* values, INN had difficulty in identifying both sources. However, at all intermediate *h* values, INN was able to resolve both sources accurately. We also explored different values of SNR and found that the results were similar to the SNR = 3 case. That is, changing SNR had no influence on the rules of selecting *h.*

### 6. Effect of the Selected Noise Subspace Dimension

Since we do not know dimensions of the noise and signal subspaces when analyzing experimental MEG data, we performed a simulation to examine the effect of the selected DIMension of the noise subspace (DIM). The simulation parameters were the same as in Section 3.2. SNR was set to 2 or 3. Two sources were correlated at *r* ^2^ = 0.90, 0.50, or 0.30. For each pair of SNR and *r* ^2^, DIM was set to 271, 270, 269, 268, 267, 262, 232, 202, 172, 142, 112, 82, 52, or 22.

**Figure 6:**
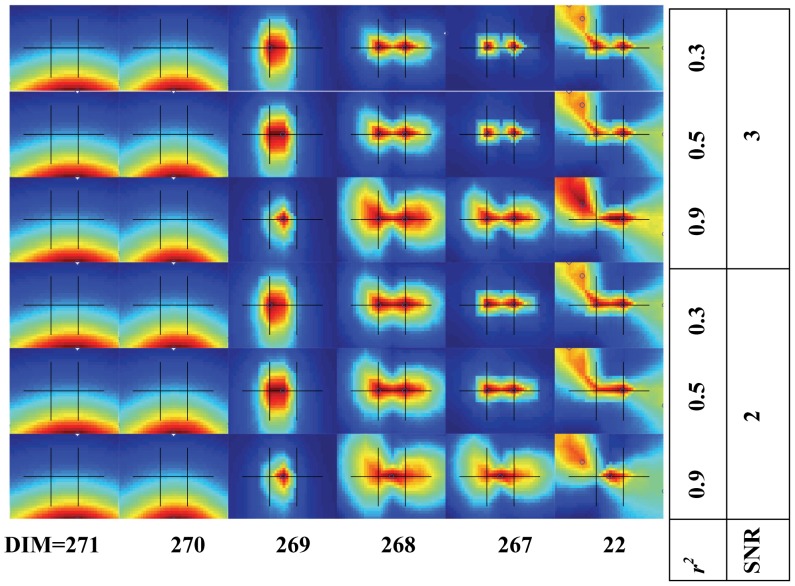
INN images for two simulated sources as a function of the dimensions of the selected noise subspace (DIM), SNR, and correlation coefficient *r* ^2^. For each fixed SNR and *r* ^2^, DIM values were between 271 (leftmost column) and 22 (rightmost column). The true source locations and the xy-plane axis scales are the same as in Figure 2 (10 mm apart). Values of SNR and *r* ^2^ are shown to the right.

Figure 6 shows the localization results using INN (*h = *1) for different DIM, SNR, and *r* ^2^ values. When DIM = 271, the noise space was overestimated, and consequently INN failed to identify both sources. At DIM = 270, the dimension of the signal subspace was 2 and INN again failed. When DIM = 269 (the dimension of signal subspace was 3), INN failed by placing a false diffused source close to the center between the two real sources. This can be explained by regarding the third left eigenvector as the transitional subspace and signal energy leaking into the noise subspace. In the case of two sources, the signal subspace should only include two left eigenvectors and the third left eigenvector should belong to noise subspace. In practice, the third eigenvector can be regarded as a transitional vector that may belong to either signal subspace (weak signal) or noise subspace (strong noise). When 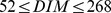, INN was able to identify the two sources. Thus, in the case of two sources, a wide range of DIM values gave reasonable results. With DIM ≤22, INN barely identified the two sources (results were very similar to those obtained when DIM = 269, not shown). In analyzing real data, the number of actual sources is unknown but typically small, suggesting that a relatively conservative DIM value should be adopted.

### 7. Performance Comparisons between the Methods

This simulation was conducted to compare the performance of INN, BEAMFORMER, MUSIC, and RAP-MUSIC. Two simulated sources were placed at (0, −60, 40) mm (“S1”) and (0, −50, 40) mm (“S2”). S1 was kept fixed whereas the *y* coordinate value of S2 was varied at *y* = −50, −45, −40, −30, −20, or −10 mm corresponding to an inter-source distance of 10, 15, 20, 30, 40, or 50 mm. We employed *r* ^2^ = 0.99, 0.7, or 0.3. For each *r* ^2^ and distance, we used SNR = 1, 1.5, 2, 2.5, 3, 4, and 5. For each combination of *r* ^2^, SNR, and inter-source distance, 100 trials were generated independently. These settings not only investigated the effect of different inter-source distance, *r* ^2^, and SNR, but also how well the method performed in the case of a superficial and a deep source (as S2 moved deeper with a larger y value). We used the mean localization bias (MLB) to quantify the localization accuracy, calculated as the average distance between the true and the estimated source locations over all the trials in the simulation.

**Figure 7:**
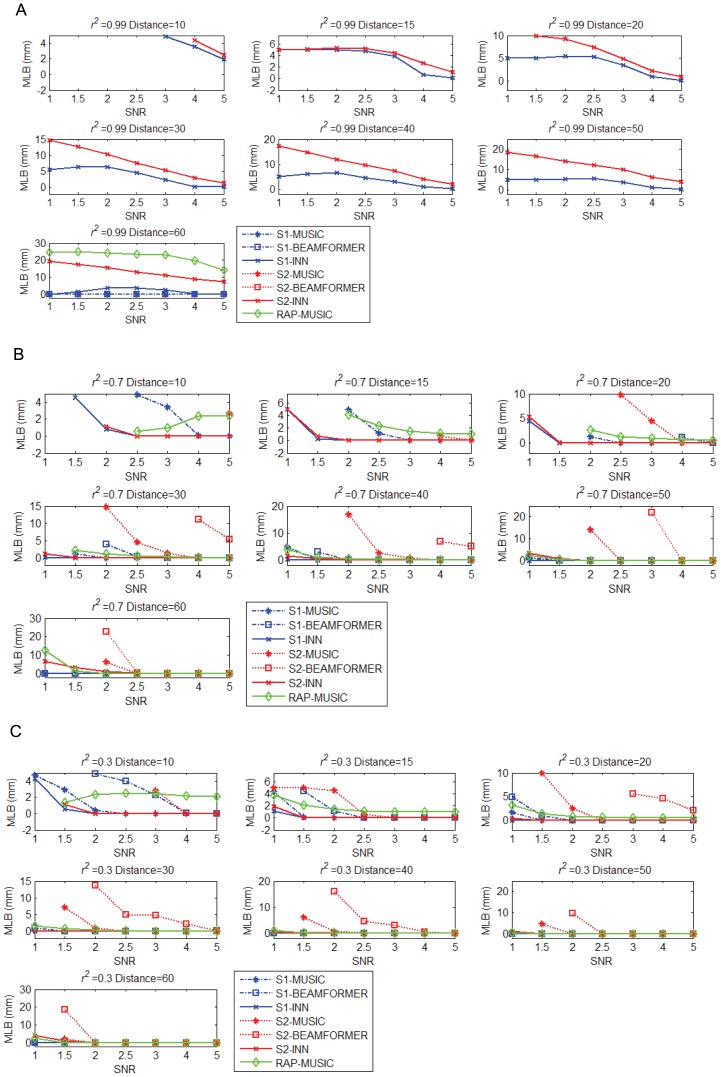
Mean Localization Bias (MLB) of two simulated sources as a function of intersource correlation r2, intersource distance, and SNR, separately for MUSIC, RAP-MUSIC, BEAMFORMER, and INN. r2 is fixed at 0.99 (a), 0.7 (b), and r2 = 0.3 (c). In each subplot, for each r2, SNR changes in the range [1,1.5,2,2.5,3,4,5] and distance in the range [10,15,20,30,40,50,60]. The points with MLB larger than the half distance between the two sources are not shown.

Figure 7 shows the MLB of the two simulated sources, S1 and S2, as a function of SNR, inter-source distance and *r* ^2^ using the four methods. Figure 7a shows the results obtained for *r* ^2^ = 0.99, Figure 7b for *r* ^2^ = 0.7, and Figure 7c for *r* ^2^ = 0.3 at different SNRs and inter-source distance.

As expected, all four methods had a smaller MLB for the superficial (S1) source and a larger MLB for the deep (S2) source. However, the localization error for these two sources was smaller for INN (*h* = 1) (red solid line for source S2 and blue solid line for source S1, data points indicated by ‘x’) than for BEAMFORMER (dotted lines, red for S1 and blue for S2, data points indicated by boxes) and MUSIC (dotted lines, red for S1 and blue for S2, data points indicated by stars). INN was the least sensitive to source depth. However, BEAMFORMER or MUSIC identified the superficial source better than the deep source, in the sense that the superficial source was identified with a smaller MLB and a much lower SNR (Figure 7b, c).

In the case of *r* ^2^ = 0.99 (Figure 7a), INN could identify the two sources under proper SNR and inter-source distance, whereas BEAMFORMER and MUSIC failed at almost all cases (except for distance = 60, where the superficial source S1 was identified accurately by BEAMFORMER and MUSIC). INN was not as sensitive to *r* ^2^, intersource distance, and SNR, as BEAMFORMER and MUSIC.

The localization performance of RAP-MUSIC (2nd recursion) depended on the accuracy of identifying the first source by MUSIC. The superficial source S1 was easier to identify and therefore, the cost function of MUSIC reached the global maximum at S1. RAP-MUSIC (2nd recursion) took S1 as the source already identified and localized source S2. As expected, RAP-MUSIC localized S2 with a smaller MLB than MUSIC.

### Real Auditory Evoked MEG Responses

Auditory evoked responses were acquired from eight healthy adult subjects with informed consent. The presented data are a subset of those previously reported in [39] where the MEG source localizations were computed using noise-normalized MNE and confirmed with fMRI in the same subjects. The stimuli were 300 ms white noise bursts and checkerboards with 300 ms duration. They were presented in a sequence consisting of auditory only (A), visual only (V), or audiovisual (simultaneous auditory and visual, AV) presentation. Stimulus events had a mean interstimulus interval of 6.1 seconds, and A/V/AV events occurred in a pseudorandom order. In the present study, we only used the responses to the auditory only (A) stimuli. Simple noise burst stimuli (such as those employed here) are known to mainly activate the supratemporal auditory cortices bilaterally [40]. Whole-head 306-channel MEG was recorded with a VectorView neuromagnetometer (Elekta Neuromag, Finland) between 0.01–330 Hz and sampled at 1 kHz. Responses from 80 trials were averaged with respect to the stimulus onset. Epochs containing electro-oculogram (EOG) signals exceeding 150 µV peak-to-peak amplitude were automatically discarded from the averages.

A three-dimensional realistically shaped volume source space with 7 mm spacing was generated using our MNE toolbox (http://www.nmr.mgh.harvard.edu/martinos/userInfo/data/sofMNE.php). Inner skull surface was extracted from a T1-weighted MRI of each subject segmented with FreeSurfer (http://surfer.nmr.mgh.harvard.edu), and used as the boundary surface for the source space. The source localization was done on the whole brain volume and the 7 mm spacing denotes the distance between grid points of a regular 3D grid defined in the brain volume. The time window for source analysis was from 0 ms to 300 ms, which covered the main response components. Source analysis was done directly on the average response.

**Figure 8:**
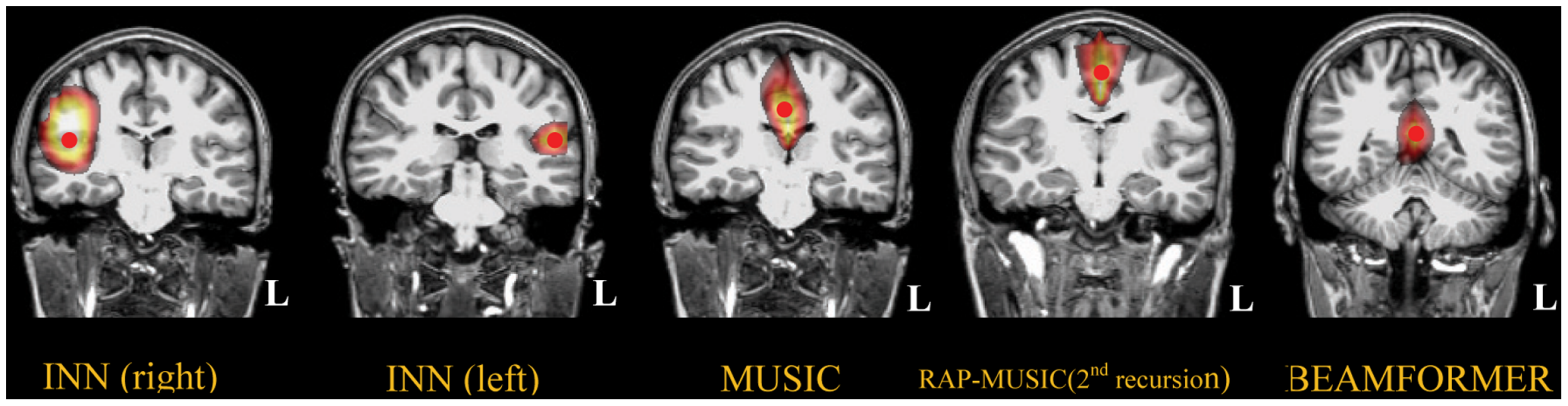
Analysis of Class 1 real auditory evoked responses using the different methods. The red points indicate the peaks of the cost functions. INN identified sources at the left and right supratemporal cortices, in agreement with locations of the auditory cortices. The threshold was set to 80% of peak of cost function within the corresponding source region. MUSIC, BEAMFORMER and RAP-MUSIC (2^nd^ recursion) found a single peak located at midline.

**Figure 9:**
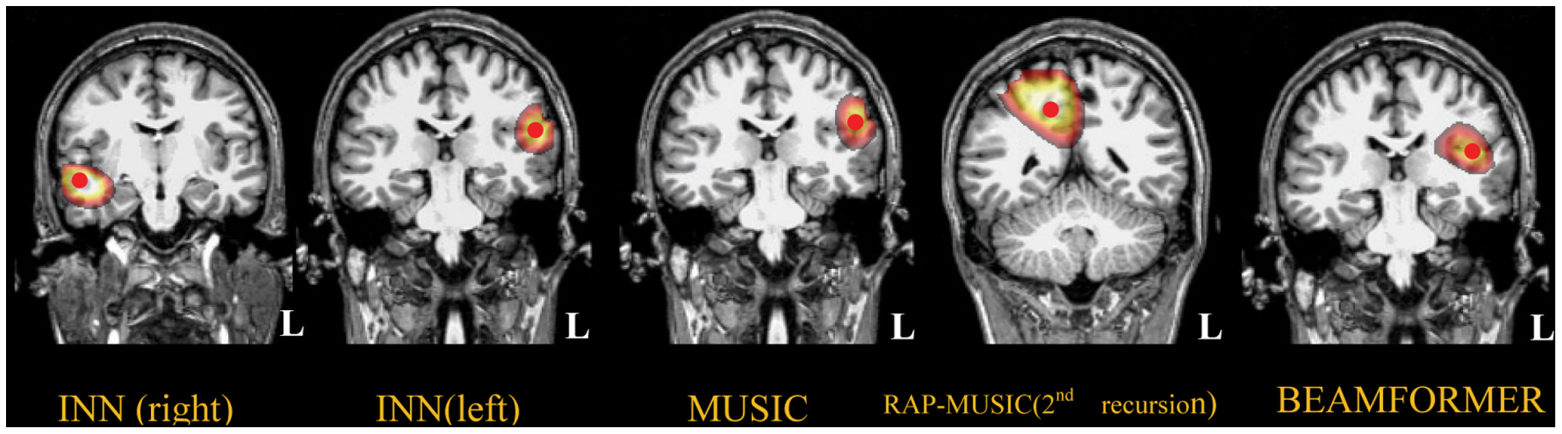
Localization of Class 2 real auditory evoked responses using the different methods. The red points indicate the peaks of the cost functions. The threshold was set to 80% of peak of the cost function within the corresponding source region. To show the underlying anatomical structure, the transparency of the overlaid images was set to 50%. INN identified sources at the left and right supratemporal auditory cortices. MUSIC and BEAMFORMER only detected a source in the left auditory cortex. RAP-MUSIC (2^nd^ recursion) misplaced a false source at midline.

**Figure 10:**
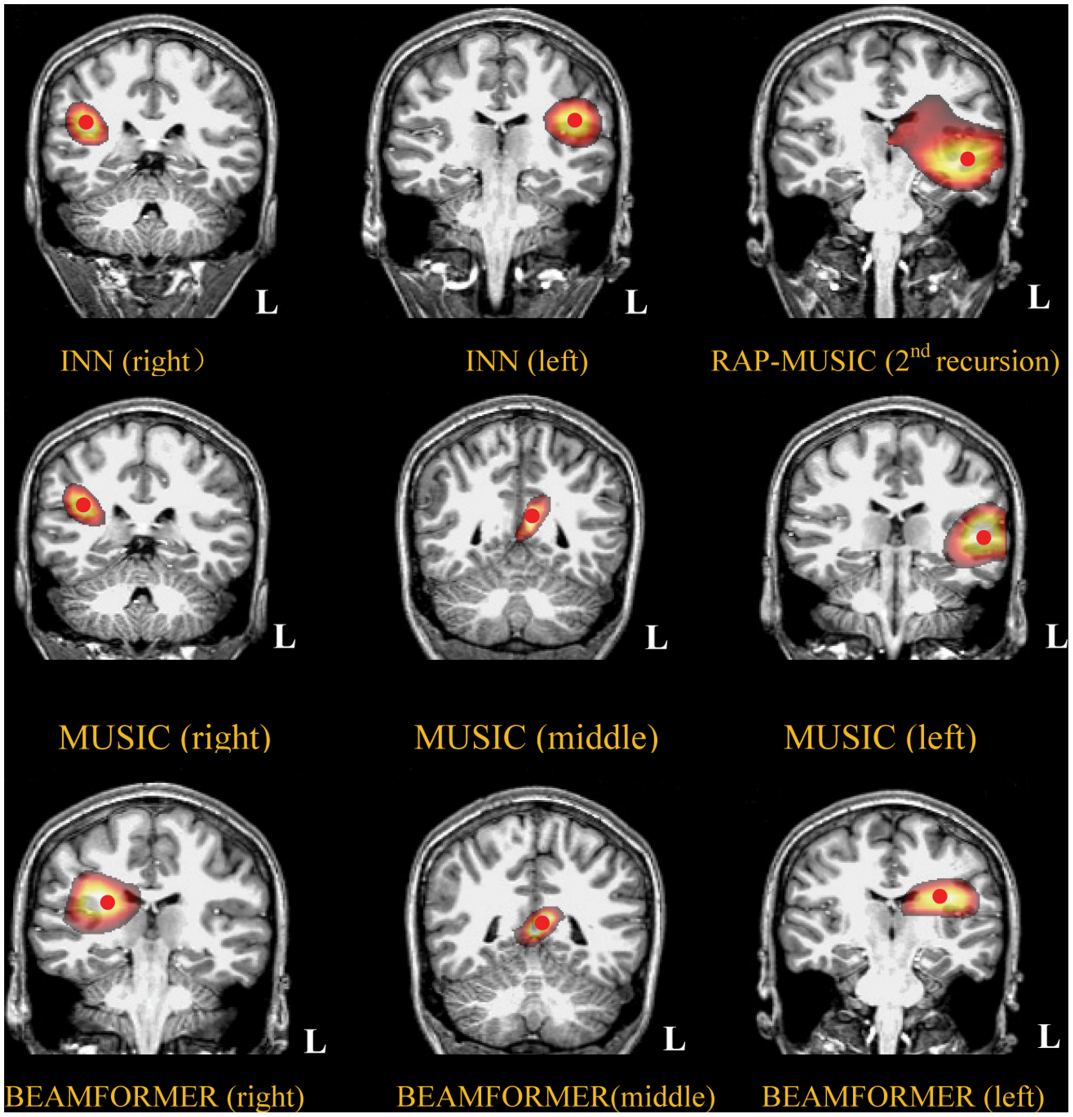
Localization of Class 3 real auditory evoked responses using different methods. The red points indicate the peaks of the cost functions. The threshold was set to 80% of peak of the cost function within the corresponding source region. To show the underlying anatomical structure, the transparency of the overlaid images was set to 50%. MUSIC found supratemporal sources in both hemispheres but also an additional spurious source in the midline. For MUSIC, the right hemisphere source was strongest (normalized maximum cost function value = 1), followed by the midline (0.83) and left hemisphere (0.63) sources. BEAMFORMER found bilateral sources that were rather deep in white matter and an additional spurious source in midline. RAP-MUSIC (2^nd^ recursion) found one source in the left temporal lobe since the right hemisphere source was suppressed. Again, INN identifed sources at the left and right supratemporal auditory cortices, in agreement with previous knowledge.

INN identified the left and right auditory cortices successfully in all 8 subjects, whereas MUSIC and BEAMFORMER failed in most occasions. Based on the type of failure, *i.e.,* the number and location of the estimated sources obtained by MUSIC and BEAMFORMER, the imaging results were divided into 3 Classes; Figures 8, 9, 10 show the corresponding results from one representative subject in each Class. For Class 1 (4 subjects, Figure 8), MUSIC and BEAMFORMER misplaced a single spurious source at midline. For Class 2 (3 subjects, Figure 9), MUSIC and BEAMFORMER only identified the left source and missed the right one. In Class 3 (1 subject, Figure 10), MUSIC and BEAMFORMER detected 3 sources, one source at the mid-sagittal plane and the other 2 sources at right and left temporal lobes (Top and middle rows in Figure 10).

## Discussion

This study introduces a novel method, INN, for MEG source localization. The basic idea is that for multidimensional spatio-temporal signals, the noise remains unchanged when the source amplitudes change. For each putative source location, INN adds a rank-one correlation matrix (obtained from the field distribution of a dipolar source) to the correlation matrix **R** of the data. At a true source location, the INN cost function, Eq.(15), will, in theory, diverge. In all other locations, the structure of signal and noise subspaces (including the spatial distribution of signal strength) will change and, therefore, the cost function will attain a smaller value. Hence, when scanning through the source space, a peak suggests a true source location.

### 1. INN is Effective for Highly Correlated Sources

Highly correlated sources are frequently encountered in MEG/EEG studies. High inter-source correlation can seriously degrade the performance of localization accuracy. Let us consider an extreme case where *r^2^* = 1, that is 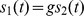. Thus, the sum of the field produced by the two sources will be 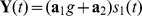 (Coherent Source Model 1, CSM1). The source correlation matrix will be proportional to 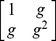, which has only rank = 1. In this case, methods based on the second order statistics such as BEAMFORMER and MUSIC usually find a spurious source whose signal pattern is close to 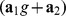 instead of 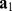 or 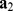. In reality, such extreme conditions are rare [30]. Therefore, depending on the source-to-source correlations, source localization results vary between complete failure identifying a source in an incorrect location and successful identification of the true sources.

In order to explain Class 3 failure of MUSIC and BEAMFORMER for the real MEG data, we can model the coherent source problem in an alternative way. Assuming two sources are highly but not fully correlated, they should have a common component 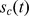. For source 1, the waveform will be the combination of the common component 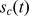 and a small independent component 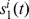, that is, 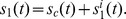 Similarly, 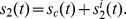 The MEG recordings will be 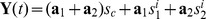 (Coherent Source Model 2, CSM2). Here, the waveforms 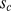, 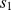, and 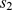 are not correlated and thus, the problem of coherent source localization is transformed into a problem involving three uncorrelated sources. Under ideal conditions, methods based on the second order statistics of the measurements 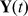 usually should be able to detect three sources, whose signal patterns are closest to 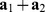, 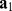, and 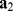 (where the signal pattern corresponding to 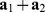 creates a spurious source between 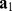, and 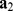). When processing real data, due to the variation of SNR, model parameters, source configuration, and possible other factors, MUSIC and BEAMFORMER may only detect the spurious source and miss the two true sources. In other cases, with appropriate parameters (*e.g.,* the remaining source component 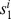 or 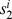 is strong enough, and SNR is proper), the two real sources may appear with smaller “power”.

INN handles correlated sources in a novel way. MUSIC or RAP-MUSIC identify sources by calculating the minimum/maximum of subspace correlation of lead field vectors at each possible source locations and noise/signal subspace of data correlation matrix. Different from MUSIC and RAP-MUSIC, INN identifies sources by comparing the sum of eigenvalue differences in noise subspace between modified matrix and the original one at each potential source location (for a detailed theoretical analysis about the difference between MUSIC and INN, please refer to the Supporting Information S1). In short, INN only utilizes the noise related eigenvalues to accomplish source localization. Therefore, the correlation between sources theoretically hardly influences the resolution characteristics of INN. Thus, as long as the sources are not fully correlated (*r* ^2^<1), INN can identify them under reasonable conditions, *i.e.,* sufficient SNR and inter-source distance.

As shown above, MUSIC generally places a false source between two closely spaced and highly correlated sources. RAP-MUSIC depends on the accuracy of MUSIC in finding the first maximum, which is then suppressed in the subsequent steps of RAP-MUSIC. Therefore, RAP-MUSIC fails if MUSIC cannot identify the location of the first source correctly. INN has considerable advantages over both MUSIC and RAP-MUSIC. Even when the sources are very close to each other, highly correlated, and noise level is high, INN still identifies the two sources correctly (see Figure 2). Moreover, as shown in Figure 3, for highly correlated but spatially distant sources, the performance of the other three methods is worse than that of INN.

### 2. INN is Effective for Sources of Different Strengths

Our simulations also suggest that INN outperforms MUSIC when there is a large strength difference between two sources. This follows from that INN only considers the variation of the noise variance distribution. In theory, the variation of the strength ratio between two sources only influences the variance distribution of the signal subspace and hardly influences that of the noise space. In cases of two sources with large relative strength difference, MUSIC may only identify the stronger source and miss the weaker one. RAP-MUSIC can handle this case readily by suppressing the source components already found. When the stronger source (found in the first MUSIC recursion) is suppressed, RAP-MUSIC will typically find the weak one in the next recursion. In this case INN and RAP-MUSIC perform equally well. However, as shown in Figure 7, where RAP-MUSIC fails for very highly correlated sources, INN still works.

Although, under suitable SNR, INN can localize sources with large differences in strength, it cannot directly recover source amplitudes or relative strengths between sources (the noise space is independent of source strengths). However, after the source locations have been identified with INN, the corresponding time courses and amplitudes can be readily extracted with other methods (*e.g.,* generalized least squares).

### 3. INN is Robust for a Wide Range of *h* and DIM Values

INN requires choosing two parameters 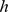 (Eq. 10) and the noise subspace dimension (DIM). Our simulations suggest that the proper values for 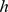 will maximize INN performance, and that INN performance is stable across a wide range of *h* values. Theoretically, any *h*>0 is sufficient for source estimation [34]. The role of *h* is to balance the order of magnitude between 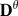 and 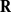. One possible option of selecting *h* is to set *h* = 1, in which case, *h* balances the order of magnitude of first term 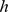 and the second factor of last term 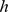
of the right side in Equation (12). In our simulations any *h* value between 1 and 10^5^ gave highly similar results. For simplicity and consistency, in the present study we used *h* = 1 in all simulations and analysis of the real data.

DIM should be selected in a range 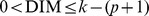 (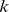 denotes the number of sensors), assuming the SVD of data correlation matrix clearly shows the signal subspace to be rank 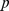. Like MUSIC or RAP-MUSIC [11], INN is also insensitive to slight overestimation of the signal subspace. It is worthwhile to note that INN fails when DIM is equal to (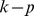). The (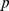
+1)^th^ eigenvector is a transitional one. Therefore, including the (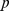
+1)^th^ eigenvector in noise subspace will deteriorate localization accuracy. In a suitable range, decreasing DIM will slightly increase the performance of INN by showing sharper peaks, since, as DIM descreases, the signal components less and less leak into the noise subspace and thus, the noise subspace can more and more sufficiently represents the noise components. The key idea of INN is to utilize some property of the noise subspace and therefore, more sufficiently representing noise components results in better localization performance for INN. Of course, DIM should not be too small, as this would result in failure due to poor representation of the noise subspace. Based on our simulations, it is better to underestimate than overestimate DIM. If the number of the sources is *p* and the number of sensors is 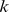, DIM should be slightly less than or equal to 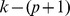.

In the present study, we mainly describe the case of two sources. For more than two sources, the selection of DIM in theory follows similar reasoning as for two sources since, generally speaking, each source is represented by one eigen component and increasing the number of sources will not change the criteria to select the noise space. However, in practice (with real MEG data) the number of sources is often unknown. If the estimated *p* is slightly larger than the real value, based on the above discussion, the performance of INN should not degrade. If the estimated *p* is smaller than the number of true sources, INN accuracy may decrease because the selected noise subspace might cover part of the signal subspace. Hence, if the number of sources is uncertain, it may be better to slightly overestimate *p* (which decreases DIM).

### 4. INN Performance with Real Data

When applied to real auditory evoked MEG responses, INN clearly outperformed the other scanning methods we evaluated, as it correctly identified the supratemporal primary auditory cortices in both hemispheres in all subjects. In the remaining part of this paragraph, we will analyze why the other methods failed. For Class 1 errors, the correlation between sources was large and SNR was low enough to almost perfectly fit CSM1. Consequently, MUSIC and BEAMFORMER only placed a combined source in the midline with its field pattern best matching that from the two bilateral sources (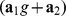 where typically 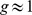), which is similar to our simulations in Figure 3 (see also [27]). Since the field pattern did not match either 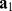 or 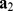, the “power” at the true source location was too small to be identified as a source (see also Eq. 11 in [41] for theoretical analysis, and real MEG data in [27], [42]). For Class 2 errors (if 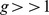
or 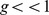 in CSM1), the combination of field patterns 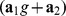 will be approximately proportional to 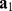 or 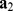 so that only one source was detected. Correspondingly, in Class 2 errors, MUSIC and BEAMFORMER only identified the left source and missed the right one. This was caused by the MEG signals over the left hemisphere being stronger than over the right hemisphere, implying that the underlying source was stronger or more superficial (see also [28]). Class 3 results can be explained using CSM2. In this case, MUSIC identified bilateral temporal sources, but along with an additional spurious source. Since the strongest MUSIC source was in the right hemisphere, RAP-MUSIC (2^nd^ recursion, after suppressing the contralateral source identified by MUSIC) identified the left hemisphere source as well, but the maximal activation was deeper than the true source. Since localization of the right hemisphere source found by MUSIC was biased, the left hemisphere source identified by RAP-MUSIC was also biased. It would be possible to improve this result by suppressing a larger region surrounding the identified right hemisphere source – this ensures that the first source is more efficiently removed even if there is a localization bias, and hence reduces problems related to inter-source correlation [42]. BEAMFORMER also identified sources in the vicinity of auditory cortices in both hemispheres and a spurious source in the midline. Based on the similar images produced in the simulated case (*e.g.,* the 3^rd^ column in Figure 3), the mid-sagittal source appears to be a spurious source resulting from the correlated bilateral auditory sources. A previous study found similar problems in most subjects [28]. In addition, due to the high inter-source correlation, both sources (one in each hemisphere) were dislocated clearly too deep in the white matter (while it is well-known that MEG/EEG signals are generated in grey matter). Although the conventional BEAMFORMER encountered difficulties in such cases, some newer variants of spatial filters might handle these situations better [27], [30], [42].

### 5. Selection of the Cost Function 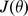


In the present study, we used the cost function specified in Eq. (15). It would be possible to modify the cost function by squaring the denominators, or multiplying them by a factor. Another option would be to substract from 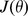 (or use it as a threshold) the theoretical value or an estimate of 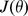 which would decrease the values in non-source locations where changes occur only in the noise subspace. Other possible modifications of Eq. (15) might be feasible as well. While such modifications might improve the source localization accuracy of INN and therefore remain an interesting topic for future work, they may also create sparser imaging results. Here, we just proposed a simple form of the cost function (the same as that in the original work) as a first approach and other options for the cost function can be explored in the future.

### Conclusions

We propose INN as a novel scanning method to identify the sources of MEG signals. We evaluated its performance for two simulated dipolar sources while varying the distance between the sources, degree of correlation between the two sources, and SNRs. We compared INN with MUSIC, conventional BEAMFORMER, and RAP-MUSIC. Based on our simulations, INN appears promising. For closely spaced correlated sources INN offers clearly better performance than MUSIC, RAP-MUSIC and BEAMFORMER. We also applied INN to human auditory evoked MEG data, where the results showed that it clearly outperforms MUSIC, RAP-MUSIC and BEAMFORMER. One shortcoming of INN, similar to other subspace-based methods, is that it cannot directly recover the amplitudes of the sources. Therefore, another approach, such as a conventional linear least-squares fit with fixed source locations and orientations, is necessary to estimate the time courses of the sources.

## Supporting Information

Figure S1The eigenvalue distribution of 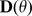 and the data correlation matrix 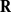. The eigenvalues are shown as function of the eigenvalue index. Two cases are illustrated: A. The orientation of the test source 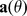 is the same as the real source when it is at a true source position (upper panel). The red lines show the distribution of new matrix 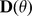 when 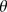 is exactly at the two true source locations. The black lines show the distribution of 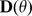 at the remaining 928 locations. The blue line shows the distribution of eigenvalues of the original correlation matrix 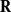. B. The orientation is not considered (directly calculate 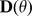 using Eq. (7)) (lower panel). Color coding is the same as in A. The simulation settings are the same as in Fig. 1 of the parent manuscript.(TIF)

Figure S2The distribution of 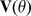 of Eq. (4). The two plots correspond to the two columns of V. Only first 10 of the 272 components are shown. The two red lines show the distributions when 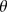 is located at each of the true source locations.(TIF)

Figure S3 The MUSIC metric with SNR = 2 shown as a function of source index (left) and its spatial distribution in the z = 40 mm plane (right). The two independent sources are correctly identified.
(TIF)

Figure S4The eigenvalue distribution of 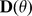 and the data correlation matrix 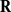. The eigenvalues are shown as function of the eigenvalue index. The red lines show the distribution of 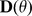 when 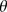 matches the two true source locations. The black lines show the distribution of 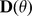 at the remaining 928 locations. The blue line shows the distribution of 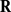. The simulation settings are the same with that in Fig. 1 of the parent manuscript. The range of the eigenvalue index is 1–10 on the left panel and 5–10 on the right panel.(TIF)

Figure S5 The INN metric with SNR = 2 as a function source index (left) and its spatial distribution in the z = 40 mm plane (right). The metric peaks at the locations of the two independent sources.
(TIF)

Figure S6The MUSIC cost function with SNR = 2 for correlated sources shown as a function source index (left) and its spatial distribution in the z = 40 mm plane (right). The locations of the sources (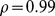) are indicated by white stars. Instead of identifying the two true sources (the two small crosses), MUSIC mistakenly detected a false source (indicated by the largest cross in the right map) placed between the two sources.(TIF)

Figure S7 The INN metric as a function source index (left) and its spatial distribution in the z = 40 mm plane (right).The metric peaks at the true locations of the two correlated sources.
(TIF)

Figure S8The eigenvalue distribution of 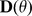 and the data correlation matrix 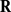. The eigenvalues are shown as a function of the eigenvalue index. The red lines show the distribution of new matrix 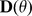 when 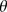 is exactly at the two true source locations. The black lines show the distribution of 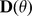 at the remaining 928 locations. The blue line shows the distribution of eigenvalues of the original correlation matrix 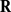. The green line shows the distribution of 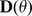 when 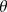 at the false source location identified by MUSIC in Fig. S6. The simulation settings are the same as in Fig. 1 of the parent manuscript. The ranges of the eigenvalue indices are 1–10 (upper panel) and 4–5 (lower panel).(TIF)

Supporting Information S1 (DOC)
