# Supplementary figures and images for: MEG Source Localization Using Invariance of Noise Space
Source: PLoS One. 2013 Mar 7;8(3):e58408. doi: 10.1371/journal.pone.0058408 (PMC3591341; doi:10.1371/journal.pone.0058408)

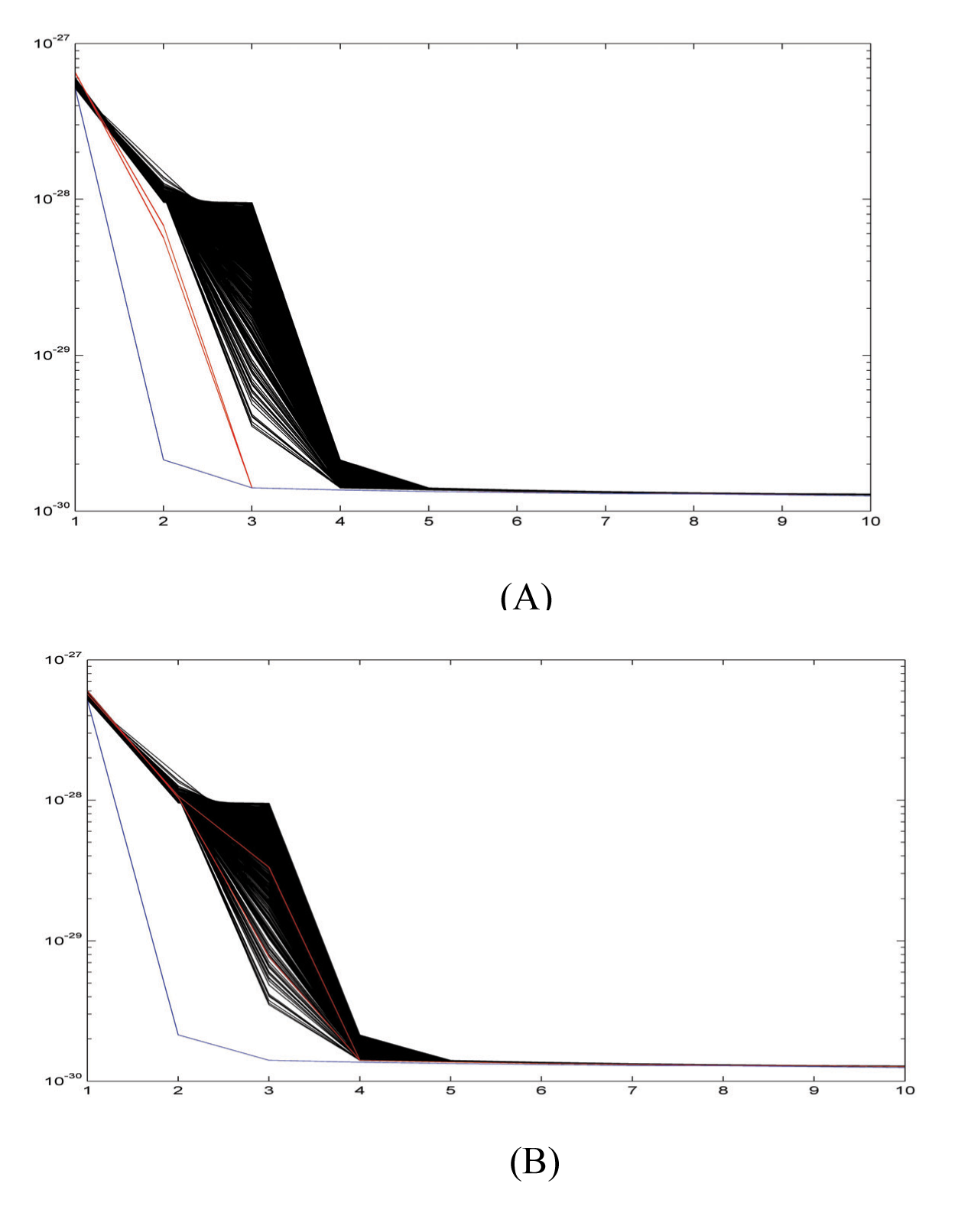

Supplement: Figure S1 — The eigenvalue distribution of and the data correlation matrix . The eigenvalues are shown as function of the eigenvalue index. Two cases are illustrated: A. The orientation of the test source is the same as the real source when it is at a true source position (upper panel). The red lines show the distribution of new matrix when is exactly at the two true source locations. The black lines show the distribution of at the remaining 928 locations. The blue line shows the distribution of eigenvalues of the original correlation matrix . B. The orientation is not considered (directly calculate using Eq. (7)) (lower panel). Color coding is the same as in A. The simulation settings are the same as in Fig. 1 of the parent manuscript. (TIF) [file pone.0058408.s001.tif]

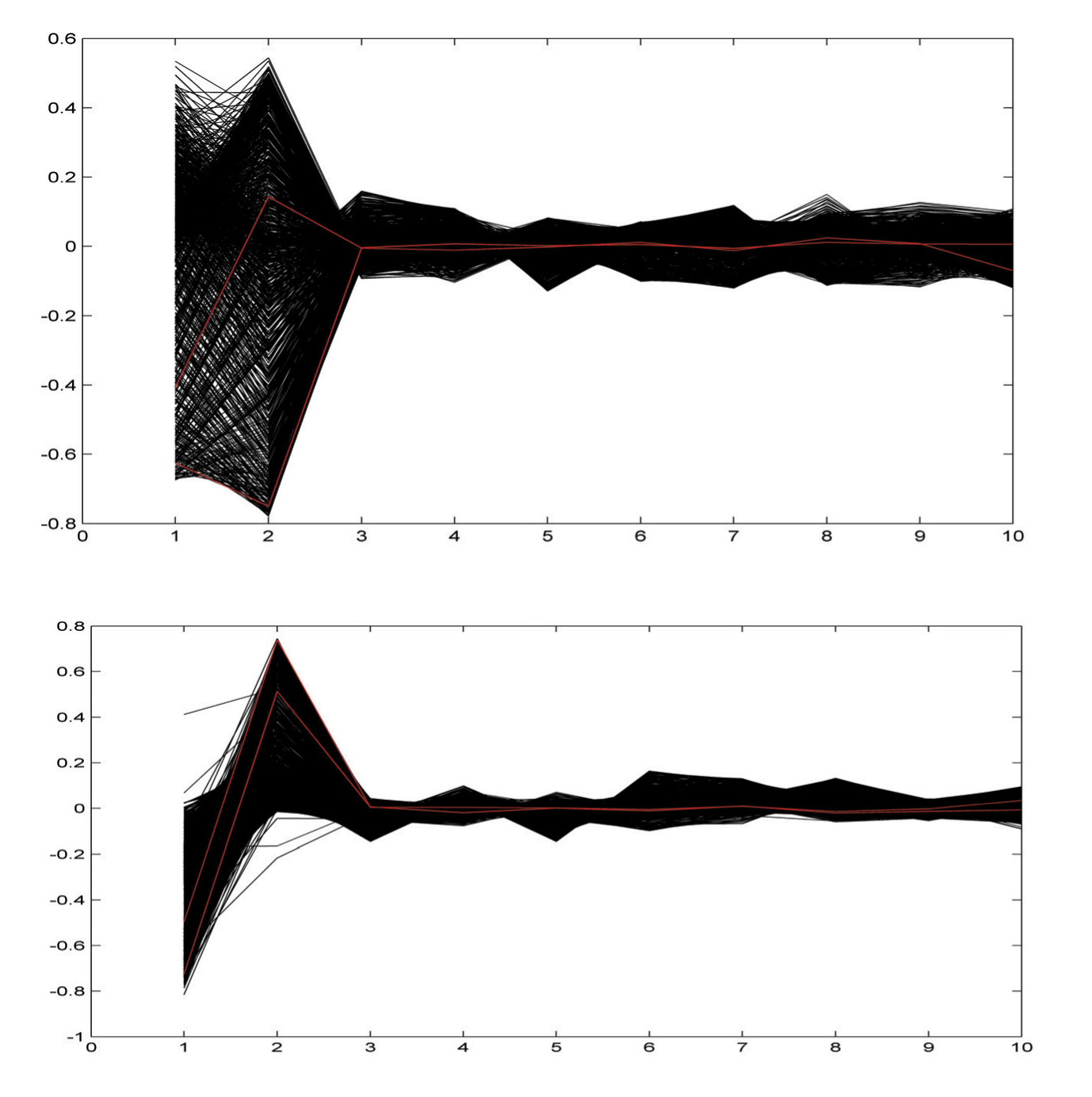

Supplement: Figure S2 — The distribution of of Eq. (4). The two plots correspond to the two columns of V. Only first 10 of the 272 components are shown. The two red lines show the distributions when is located at each of the true source locations. (TIF) [file pone.0058408.s002.tif]

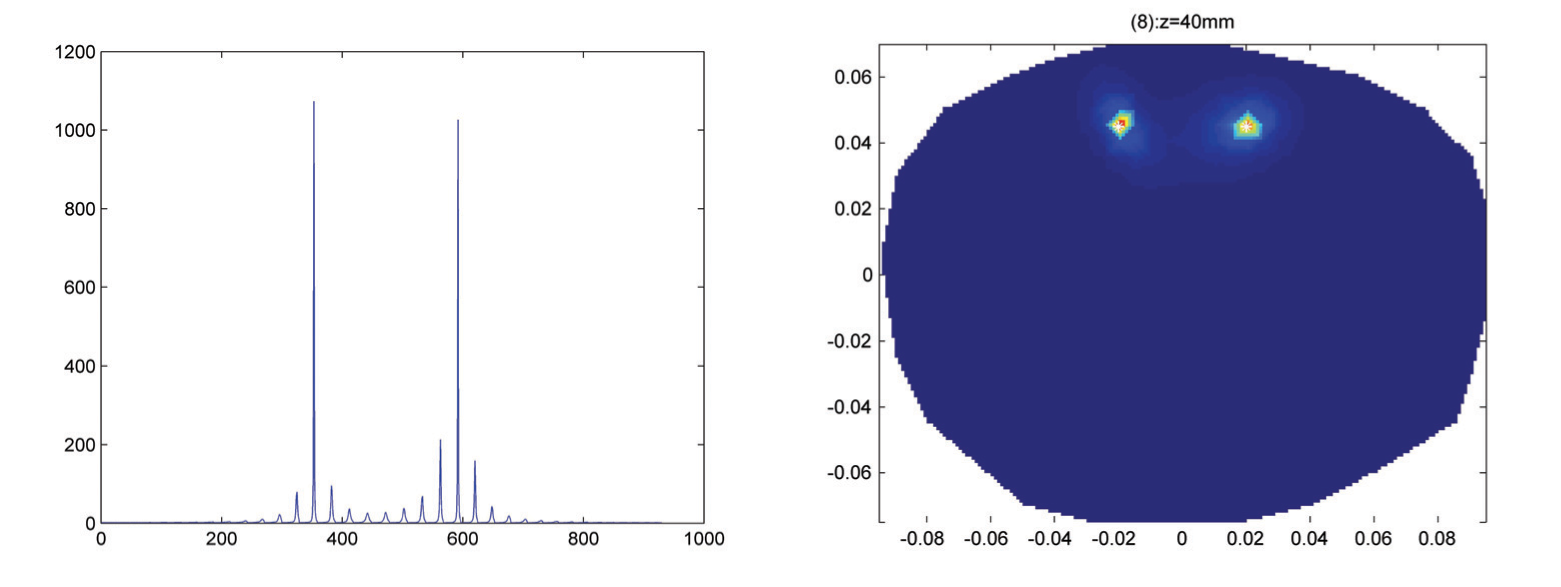

Supplement: Figure S3 — The MUSIC metric with SNR = 2 shown as a function of source index (left) and its spatial distribution in the z = 40 mm plane (right). The two independent sources are correctly identified. (TIF) [file pone.0058408.s003.tif]

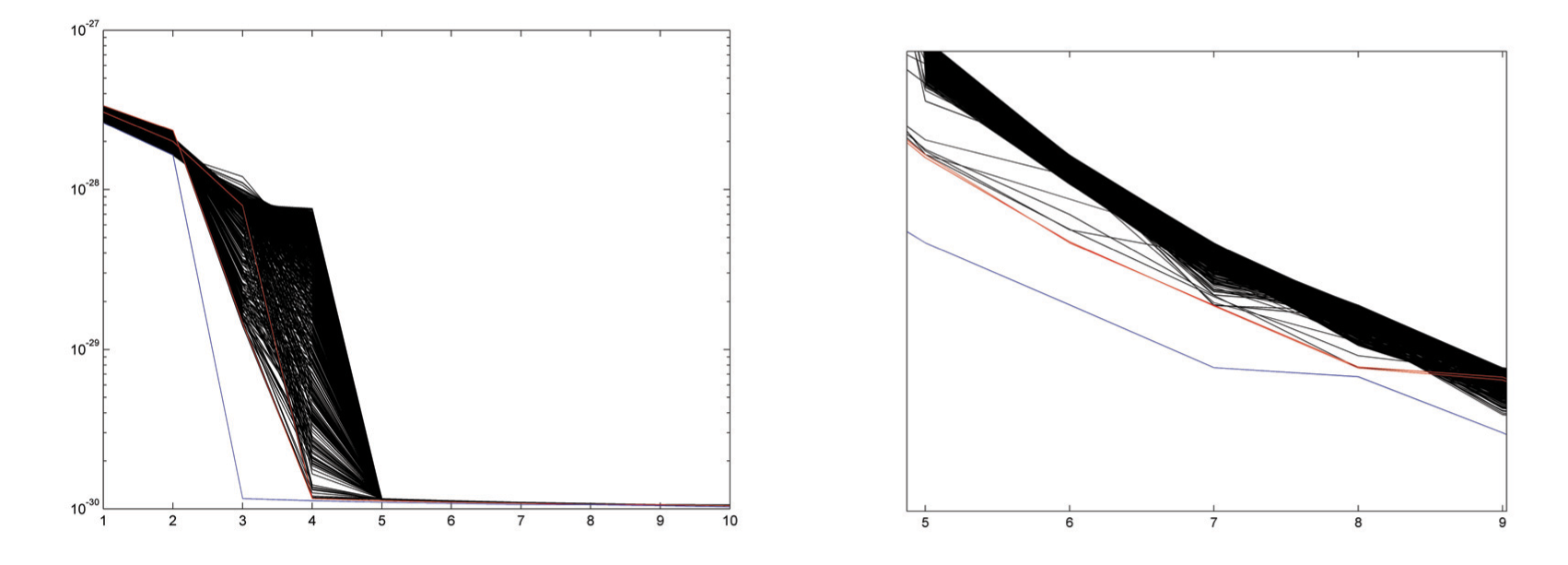

Supplement: Figure S4 — The eigenvalue distribution of and the data correlation matrix . The eigenvalues are shown as function of the eigenvalue index. The red lines show the distribution of when matches the two true source locations. The black lines show the distribution of at the remaining 928 locations. The blue line shows the distribution of . The simulation settings are the same with that in Fig. 1 of the parent manuscript. The range of the eigenvalue index is 1–10 on the left panel and 5–10 on the right panel. (TIF) [file pone.0058408.s004.tif]

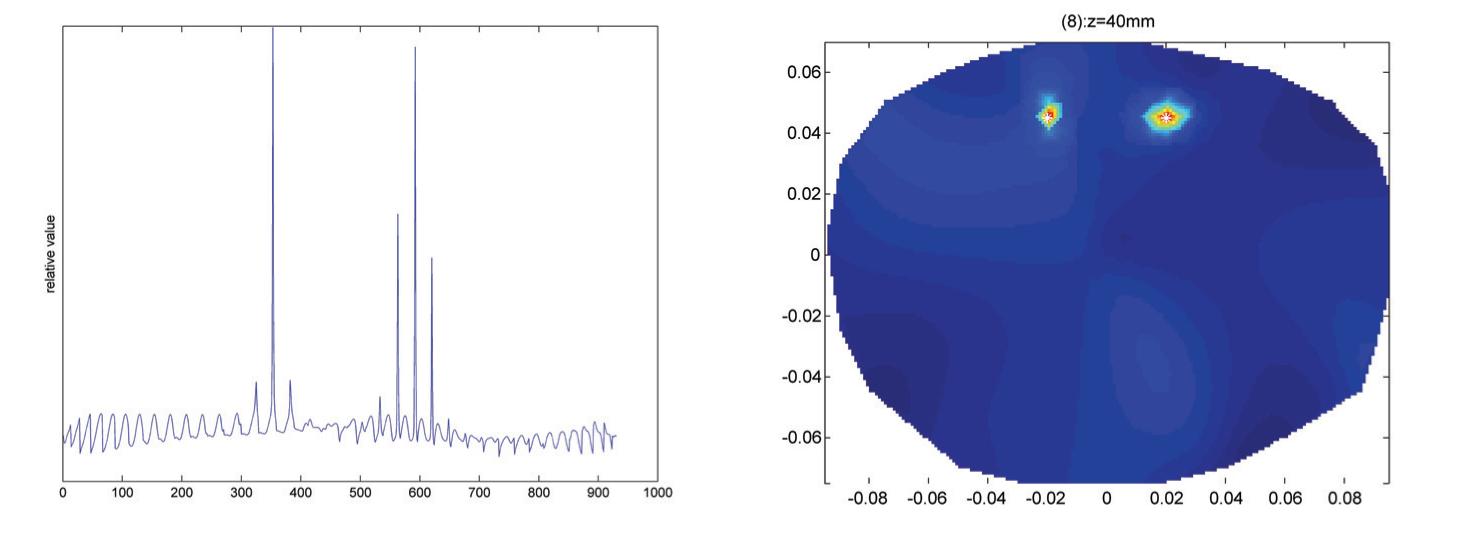

Supplement: Figure S5 — The INN metric with SNR = 2 as a function source index (left) and its spatial distribution in the z = 40 mm plane (right). The metric peaks at the locations of the two independent sources. (TIF) [file pone.0058408.s005.tif]

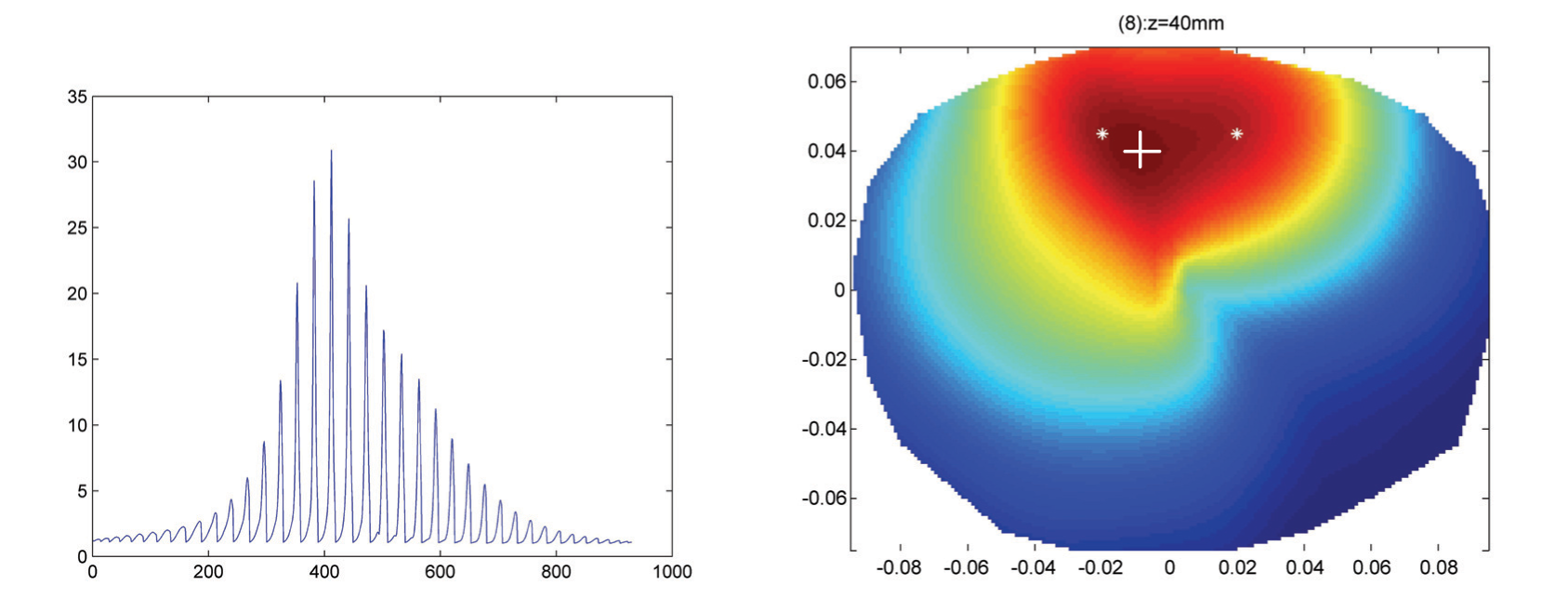

Supplement: Figure S6 — The MUSIC cost function with SNR = 2 for correlated sources shown as a function source index (left) and its spatial distribution in the z = 40 mm plane (right). The locations of the sources () are indicated by white stars. Instead of identifying the two true sources (the two small crosses), MUSIC mistakenly detected a false source (indicated by the largest cross in the right map) placed between the two sources. (TIF) [file pone.0058408.s006.tif]

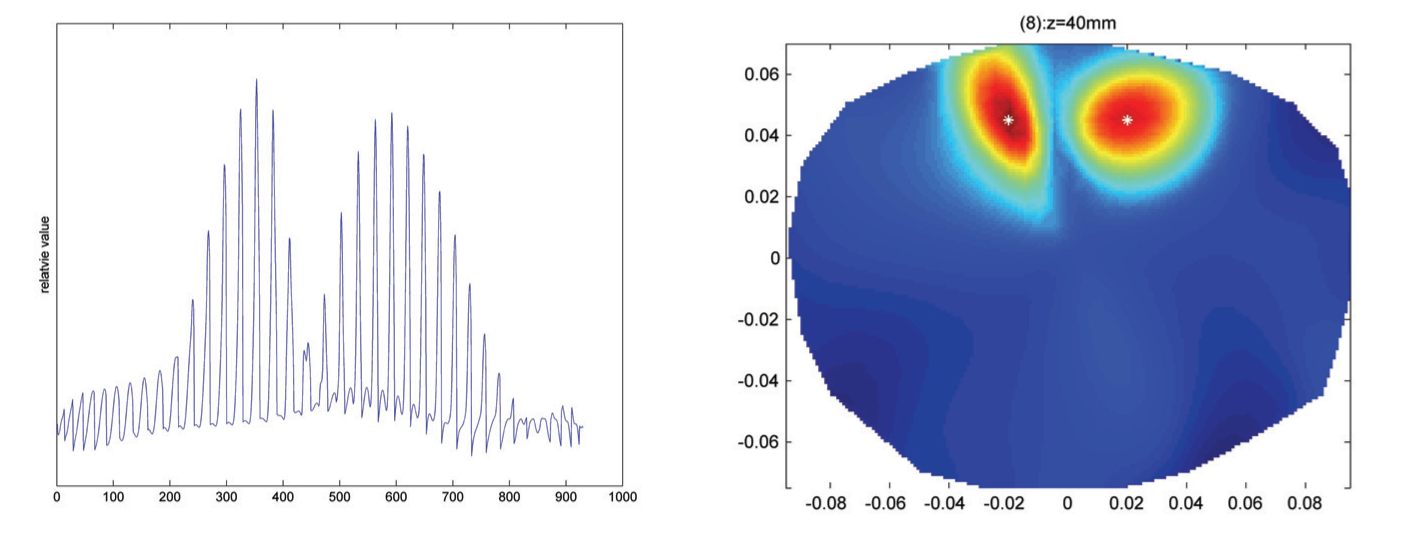

Supplement: Figure S7 — The INN metric as a function source index (left) and its spatial distribution in the z = 40 mm plane (right).The metric peaks at the true locations of the two correlated sources. (TIF) [file pone.0058408.s007.tif]

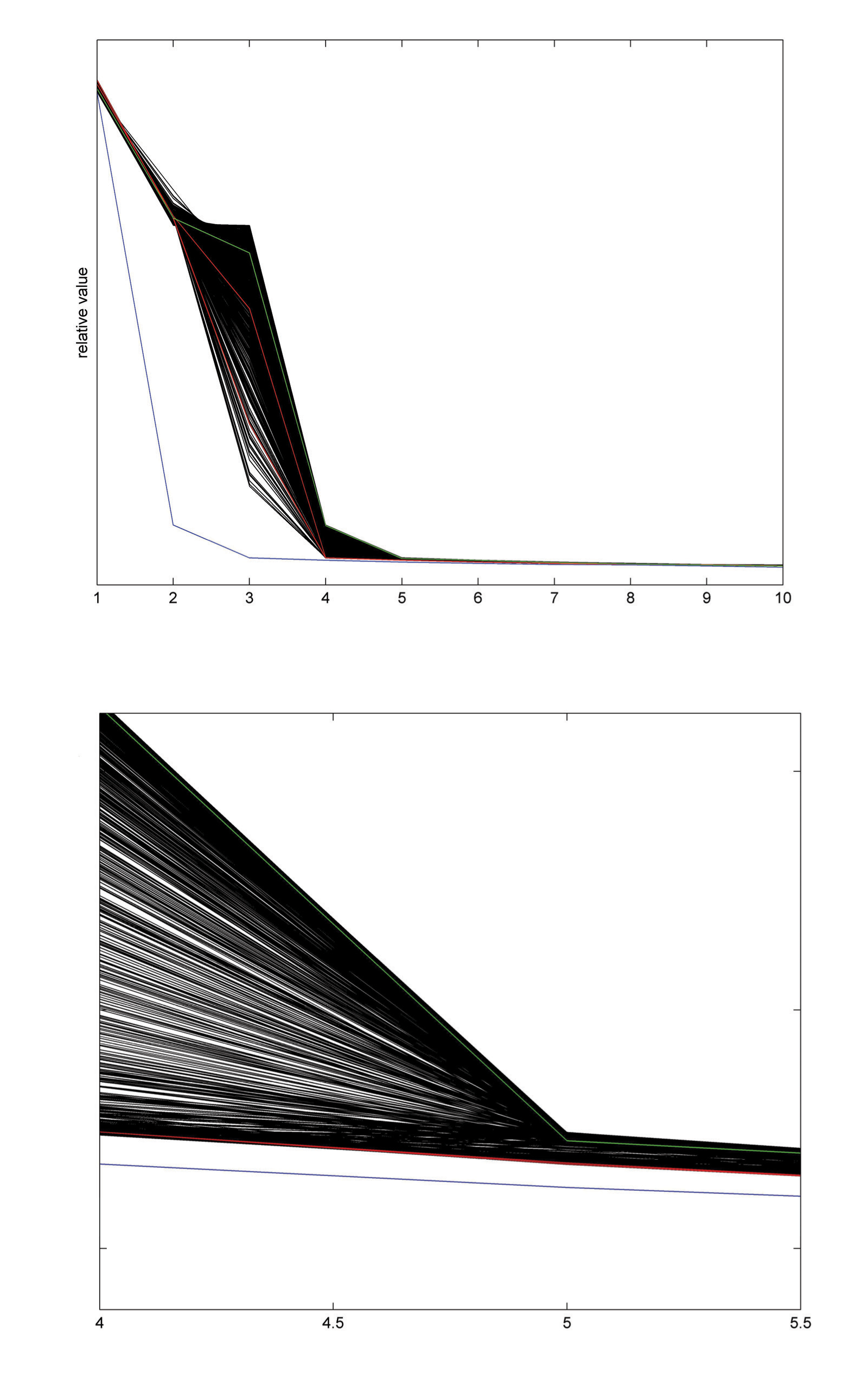

Supplement: Figure S8 — The eigenvalue distribution of and the data correlation matrix . The eigenvalues are shown as a function of the eigenvalue index. The red lines show the distribution of new matrix when is exactly at the two true source locations. The black lines show the distribution of at the remaining 928 locations. The blue line shows the distribution of eigenvalues of the original correlation matrix . The green line shows the distribution of when at the false source location identified by MUSIC in Fig. S6. The simulation settings are the same as in Fig. 1 of the parent manuscript. The ranges of the eigenvalue indices are 1–10 (upper panel) and 4–5 (lower panel). (TIF) [file pone.0058408.s008.tif]
