# Supplementary material for: MEG Source Localization Using Invariance of Noise Space
Source: PLoS One. 2013 Mar 7;8(3):e58408. doi: 10.1371/journal.pone.0058408 (PMC3591341; doi:10.1371/journal.pone.0058408)
Supplement: Supporting Information S1 — (DOC) [file pone.0058408.s009.doc]

**The difference between MUSIC and INN**

**1. Notation definition**

An estimate of the data covariance matrix $\mathbf{R}$ can be constructed from the time series of MEG measurements **Y**, where **Y** is a matrix of ‘*n by* *t’* including *n*-channel MEG measurements across *t* time points:

$\mathbf{R}=\mathbf{Y'Y}/n$ (S1)

Using the eigenvalue decomposition of **R** we can define the ‘signal subspace’ and the ‘noise subspace’, which are spanned by columns of **E**_s_ and **E**_n_, respectively:

, (S2)

where *diag*(•) represents constructing a diagonal matrix from the vector argument. The above division assumes that the largest *p* eigenvalues and eigenvectors are associated with the signal subspace and the remaining (*n*-*p*) eigenvalues and eigenvectors with the noise subspace, respectively.

At a given source location, we use a $n\times2$ matrix $\mathbf{a}\left(\theta\right)$ to specify the theoretical measurements by MEG channels when the source is a current dipole at location given by *θ* with two orthogonal orientations (a sphere model for MEG is used here).

The amplitude of **a**(*θ*) can be normalized

$\mathbf{a}\left(\theta\right)=\mathbf{u}\left(\theta\right)\mathbf{k}\text{ },\text{ }\mathbf{u}\left(\theta\right)^{T}\mathbf{u}\left(\theta\right)=\mathbf{I}$ (S3)

where **k** is a diagonal matrix used to normalize the gain matrix $\mathbf{a}\left(\theta\right)$**.** I is a unit matrix.

With a full-rank **E**, **a**(*θ*) can also be projected in the columns of **E**:

$\mathbf{u}\left(\theta\right)=\mathbf{EV}\left(\theta\right)=\left[\mathbf{E}_{s}\text{ }\mathbf{E}_{n}\right]\left[\begin{matrix} \mathbf{v}_{s}\left(\theta\right) \\ \mathbf{v}_{n}\left(\theta\right) \end{matrix}\right]\text{ }$, (S4)

where *v*_s_ and *v*_n_ are *p* x 2 and *(n-p)* x 2 matrices of the expansion coefficients.

**2. MUSIC**

In MUSIC one evaluates

$\eta_{MUSIC}\left(\theta\right)=\lambda_{max}\{\mathbf{u}^{T}(\theta)\mathbf{E}_{s}\mathbf{E}_{s}^{T}\mathbf{u}(\theta)\}$ (S5)

across different source locations *θ* . Using the orthogonal property between signal and noise subspaces and Eq. (S4), η_MUSIC_(*θ*) can be written as

$\eta_{MUSIC}\left(\theta\right)=\lambda_{max}\{\mathbf{u}^{T}(\theta)\mathbf{E}_{s}\mathbf{E}_{s}^{T}\mathbf{u}(\theta)\text{\} =}\lambda_{max}\{\mathbf{v}_{s}^{T}(\theta)\mathbf{v}_{s}(\theta)\}$. (S6)

1. **INN**

In INN, one constructs a new matrix for each position: the original data correlation matrix **R** plus a position specific matrix.

(S7)

$\left[\Lambda+hk^{2}\frac{trace(\mathbf{R})}{trace(\mathbf{a}(\theta)*\mathbf{a}(\theta)^{T})}\mathbf{V}(\theta)\mathbf{V}^{T}(\theta)\right]$ is not a diagonal matrix and thus, $\mathbf{E}_{new}$is different from the matrix of the eigenvectors of **R** employed in MUSIC. Therefore, both the eigenvalues and eigenvectors of $\mathbf{D}(\theta)$ are different from those of **R**.

The metric of INN, as defined in the manuscript, is

$\eta_{INN}\left(\theta\right)=1/\left(\sum_{i=p+2}^{n} \left(\lambda_{INN}^{i}-\lambda^{i}\right)\right)$. (S8)

If we set the number of sources or the dimension of the signal subspace to *p*, in most cases, the dimension of signal subspace of $\mathbf{D}(\theta)$ should be *p+1* (if the newly added source is exactly at one of the true source locations, the dimension of the signal subspace of $\mathbf{D}(\theta)$ still is *p*). INN focuses on noise space invariance. Therefore, the noise subspace dimension, which is used for calculating the cost function of INN, should be *n-(p+1)* (from *p+2* to *n*); we make this assumption in all our calculations.

1. **INN and MUSIC**

The crucial differences between INN and MUSIC are:

1. In MUSIC, the signal subspace and noise subspace are fully determined by the original data correlation matrix **R**. The sources are found based on the correlation between the gain matrix $\mathbf{a}\left(\theta\right)$ and the signal subspace or, equivalently, the orthogonality between the gain matrix $\mathbf{a}\left(\theta\right)$ and noise subspace.
2. In INN, the signal subspace and noise subspace are recalculated for each probe source location. Therefore, eigenvalue distribution may be different for different probe source locations, as confirmed by our simulations.

In fact, we found that when the position and orientation of a test vector /matrix $\mathbf{a}\left(\theta\right)$ match those of one of the true sources, the change of eigenvalue distribution will be constrained in the signal subspace whereas the eigenvalue in the noise subspace stays unchanged (invariance of noise space) (see Fig.1). It is this fact that provides us a “maximum/minimum point” of the cost function to find the source position by observing changes of the eigenvalues in the noise subspace over the total solution space. Thus, we use this change as the imaging measure/index to perform source localization.

In summary, **INN is sensitive to the change of eigenvalues caused by the probe source $\mathbf{a}(\theta)$, while MUSIC compares the gain matrix of the probe source to a fixed signal/noise subspace estimated from the recorded data**. This difference makes it possible for INN to resolve some cases which are problematic for MUSIC, as confirmed by our simulations in the manuscript and below.

1. **Simulations and theoretical analysis**

**Simulation 1: Illustration of the principle**

Two independent sources (at voxel indices 353 and 594) are located at z=40 mm plane, SNR=2. Other model settings are the same as in Section 3.1 of the manuscript.

When the location and orientation of the test vectors $\mathbf{a}(\theta)$ matches those of the two true sources (here we assume the locations and orientations to be known), the eigenvalue distributions of $\mathbf{D}(\theta)$ and **R** are as shown in Fig. S1A. If the orientation of the test vectors is not considered, *i.e.,* $\mathbf{D}(\theta)$ is calculated directly using Eq. (S7)), the distribution of $\mathbf{D}(\theta)$ and **R** are as shown in Fig. S1B. Obviously, in the former case (Fig. S1A), when the probe is at the real source location, the dimension of the signal subspace changes to two, but the noise subspace keeps unchanged. In the latter case (Fig. S1B), we did not consider the source orientation, which is usually not known. In this case, the changes occur in both the signal and noise subspaces. As shown below, we will employ the change in the noise subspace to find the source location.

**Simulation 2: Independent sources (both MUSIC and INN work well)**

**MUSIC**

For the source constellation of Simulation 1, Fig. S2 shows that most of the energy of $\mathbf{a}(\theta)$ is projected on the first two eigen vectors when $\theta$ matches the two real source locations exactly. The spatial distribution of the MUSIC metric is shown in Fig. S3.

**INN**

Fig. S4 shows the result of recalculating the eigenvalue distribution for each probe source position. The corresponding spatial distribution of the INN metric is shown in Fig. S5.

In Fig. S4, as especially visible in the zoomed part of the noise subspace (right panel), the eigenvalues corresponding to a probe source at one of the true source positions (red lines) are much closer to the blue line (the distribution of eigenvalues of $\mathbf{R}$). Furthermore, they are smaller than at the incorrect positions (black lines). This separation is used to determine the source locations.

**Simulation 3: Highly correlated sources (INN works well, MUSIC fails)**

**MUSIC**

Two highly correlated sources with correlation coefficient $\rho=0.99$ are placed at the plane z = 40 mm. Other settings are the same as in Section 3.1 above.

Fig. S6 shows the cost function distribution and imaging result obtained by MUSIC. Conventional MUSIC has difficulty in resolving highly correlated sources: the MUSIC metric peaks between the two real sources (for a theoretical analysis, please see Section 5.1 2^nd^ paragraph of the parent manuscript). At the false source location $\theta_{f}$ and those (denoted by $\theta_{1}$ and $\theta_{2}$) of the two real source locations $\mathbf{a}(\theta_{f})\approx0.7*\mathbf{a}(\theta_{1})+0.2*\mathbf{a}(\theta_{2})$: due to the high correlation between the two real sources, they were merged into one equivalent source.

The case of correlated sources could have been handled better by MUSIC if a source pair would have been used. The caveat is that the metric then depends on six parameters, three for each dipole.

**INN**

Fig. S7 shows that, with the same source and model settings as above, INN successfully identified the two sources.

Fig. S8 shows the eigenvalue distribution of $\mathbf{D}(\theta)$ at all source locations. Clearly, at true source locations, the eigen values are mixed together with the others in signal subspace (according to the top in Fig. S8, a proper choice of the dimension of signal subspace should be 3) and it is impossible to distinguish the true sources from the other locations. At the false equivalent source (misidentified by MUSIC) location, the eigen values also are mixed in the signal subspace. On the contrary, in noise subspace (eigen index from 4 to 272), the eigen values can be distinguished between 1) real source locations, 2) false source location $\theta_{f}$ and the other positions. Correspondingly, in Fig. S8 (lower panel), the red lines are slightly above the blue line and are almost always lower than all the other black and green lines (some of them with positions $\theta$ very close to the true source positions). INN utilizes these properties for localization. This example also illustrates the reason for using the eigen value change in the noise subspace instead of its change in the signal subspace. The correlated sources mainly induce signal subspace distortion.

Comparing Figs. S4 and S8 shows that increasing the correlation between sources has a negative effect on performance of INN as well: the red lines, indicating the eigenvalue distribution at the real source locations, are closer to the group of the black lines corresponding to all other locations. Although performance of INN degraded in this extreme case, the two sources were still identified successfully.

We also conducted simulations in the cases of (i) two closely spaced sources, and (ii) two sources with large amplitude differences (see the parent manuscript). In both cases INN was superior to MUSIC.

Comparing Eq. (S6) with Eq. (S8), it becomes evident that the cost function of MUSIC and INN are based on different key ideas. MUSIC uses the largest subspace correlation between the gain matrix and the signal subspace to find the sources, whereas INN considers the difference of eigenvalues in noise subspace between the modified matrix and the original data correlation matrix.

MUSIC focuses on the subspace correlation between signal subspace and the gain vector/matrix of the candidate sources or, equivalently, their orthogonality to the noise subspace.

INN focuses on the eigenvalue distribution difference, only considering those lying in the noise subspace, between $\mathbf{D}(\theta)$ and the original **R**. Apparently, INN relaxes the requirement of a correct signal/noise subspace identification required by MUSIC. It pays attention to the eigenvalue distribution change in the noise subspace, which avoids wrong signal subspace identification caused by the correlations among sources.

In conclusion, MUSIC performs well in most cases. However, MUSIC encounters difficulties in important special cases where INN can offer better performance. Generally, both MUSIC and INN stem from the array signal processing theory. They utilize different aspects of the signal *vs.* noise subspace decomposition. MUSIC utilizes the angle between the gain vectors $\mathbf{a}(\theta)$ and the signal subspace to look for sources, since at the source location, $\mathbf{a}(\theta)$ usually has a minimum angle with signal subspace (if the signal subspace is correctly recovered). That is, MUSIC focuses on the relationship of directions characterized by eigenvectors $\mathbf{u}(\theta)$ and $\mathbf{E}_{s}$. In contrast, INN focuses the signal/noise space disturbance, *i.e.*, the energy distribution disturbance in signal and noise subspace, especially the noise subspace, caused by a testing source$\mathbf{a}(\theta)$. Our results confirm the merits of INN in source localization in cases when MUSIC encounters problems.

**1. Mosher, J.C. and R.M. Leahy, *Source localization using recursively applied and projected (RAP) MUSIC.* IEEE Transactions on Signal Processing, 1999. 47(2): p. 332-340.**
